# How does spatial extent and environmental limits affect the accuracy of species richness estimates from ecological niche models? A case study with North American Pinaceae and Cactaceae

**DOI:** 10.1002/ece3.10007

**Published:** 2023-04-21

**Authors:** Mir Muhammad Nizamani, Monica Papeş, Hua‐Feng Wang, AJ Harris

**Affiliations:** ^1^ Sanya Nanfan Research Institute of Hainan University, Hainan Yazhou Bay Seed Laboratory Sanya China; ^2^ Hainan Key Laboratory for Sustainable Utilization of Tropical Bioresources, College of Tropical Crops Hainan University Haikou China; ^3^ Department of Ecology and Evolutionary Biology University of Tennessee Knoxville Tennessee USA; ^4^ South China Botanical Garden, Chinese Academy of Science Guangzhou China

**Keywords:** biodiversity, biogeography, floras, Maxent, species richness, stacked species distribution models

## Abstract

Measuring species richness at varying spatial extents can be challenging, especially at large extents where exhaustive species surveys are difficult or impossible. Our work aimed at determining the reliability of species richness estimates from stacked ecological niche models at different spatial extents for taxonomic groups with vastly different environmental dependencies and interactions. To accomplish this, we generated ecological niche models for the species of Cactaceae and Pinaceae that occur within 180 published floras from North America north of Mexico. We overlaid or stacked the resulting species’ potential distribution estimates over the bounding boxes representing each of the 180 floras to generate predictions of species richness. In general, our stacked models of Cactaceae and Pinaceae were poor predictors of species richness. The relationships between observed and predicted values improved noticeably with the size of spatial extents. However, the stacked models tended to overpredict the richness of Cactaceae and over‐ and underpredict the richness of Pinaceae. Cactaceae stacked models showed higher sensitivity and lower specificity than those for Pinaceae. We conclude that stacked ecological niche models may be somewhat poor predictors of species richness at smaller spatial extents and should be used with caution for this purpose. Perhaps more importantly, abilities to compensate for their limitations or apply corrections to their reliability may vary with taxonomic groups.

## Graphical abstract

The objective of our work was to determine the reliability of species richness estimates from stacked ecological niche models at different spatial extents for taxonomic groups that have vastly different environmental dependencies and interactions. We conclude that stacked ecological niche models may be somewhat poor predictors of species richness and should be used with caution for this purpose. Perhaps more importantly, abilities to compensate for their limitations or apply corrections to their reliability may vary with taxonomic groups.

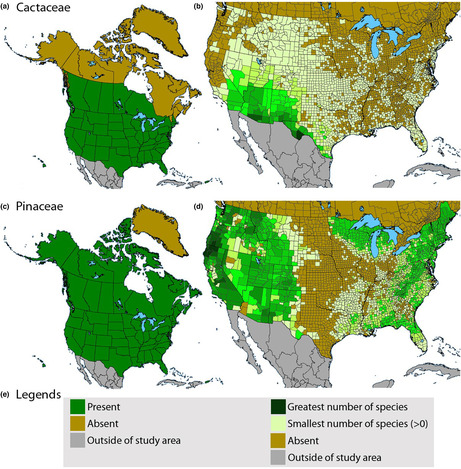

## 1 INTRODUCTION

Species richness, the number of unique species that inhabit a geographic area, represents a key component to measuring biodiversity in basic and applied ecology (Haack et al., 2021; Hillebrand et al., 2018; Lawrence & Fraser, 2020; Mitchell et al., 2020). However, species richness is challenging to measure at some spatial extents, especially larger ones for which exhaustive species surveys are difficult or impossible (Fontana et al., 2020; Lawrence & Fraser, 2020; Roswell et al., 2021). In the case of vascular plants, species richness can be tallied from the species lists in floras. A flora is an inventory of plants of an area (Palmer et al., 1995). Floras are typically the most comprehensive and authoritative resources for species richness within a geographic area (Xu et al., 2020). However, publishing floras is limited by the time‐consuming collection of data on species’ distributions and by the person‐hours and expertise required (Cardoso et al., 2017; Funk, 2006; Palmer et al., 1995; Rouhan & Gaudeul, 2014; Wen et al., 2017). While automated and partially automated use of big biodiversity data may reduce these limitations in compiling floras (Barkworth et al., 2020; Boho et al., 2020; Miller et al., 2015; Palese et al., 2019), alternatives to inferring species richness are desirable at present.

An alternative method of assessing species richness of vascular plants and other organisms relies on estimating species’ potential distributions with ecological niche models (ENMs) and overlaying or stacking the potential distributions (Biber et al., 2020; de Andrade et al., 2020; Feng et al., 2019; Grenié et al., 2020; Saunders et al., 2020). The ENMs are generated from database records of species occurrences and environmental predictors. Many local, regional, and international databases of digitized species’ occurrences have been assembled in the last couple of decades (Qian et al., 2018), and these are increasingly integrated in the Global Biodiversity Information Facility (GBIF; https://www.gbif.org/). Stacked ENMs have been widely used to estimate the species richness of differently sized geographic areas and taxonomic groups, such as herbaceous plants, woody plants (Pouteau et al., 2015), insects (D'Amen, Pradervand, & Guisan, 2015), mammals (Tobeña et al., 2016), and amphibians (Xicuo, 2016).

The reliability of ENMs as predictors of species richness is expected to vary because models can estimate species’ potential suitability rather than occupied areas and additional parameters such as species’ dispersal may be needed to approximate occupied area (Cooper & Soberón, 2018). The spatial extent of the investigation may also influence the ability to predict richness from ENMs. For example, Feria and Peterson (2002) proposed that this predictive ability would be lower at local scales where interaction effects will be more important than at regional or continental scales. Velazco et al. (2017) showed that large extents incorporate more environmental variability in ENMs that allowed for better discrimination between suitable and unsuitable environments and thus increased the accuracy of models. In a study covering Japan, Ishihama et al. (2019) showed that ENMs might be more accurate for smaller areas, of 1–5% of the country.

Plant studies, in particular, have assessed the reliability of stacked ENMs by comparing them to species lists for small, exhaustively surveyed vegetation plots (von Takach et al., 2020). Those studies have generally detected overprediction of species richness based on ENMs (Del Toro et al., 2019; Mendes et al., 2020). However, few prior studies have quantified the relationship between the sizes of geographic areas and the reliability of stacked ENMs to predict species richness. In a study of Chilean vascular flora, Luebert et al. (2022) found that richness estimated with ENMs was most reliable at coarse extents of 100 and 75 km. More studies are needed to build a broad understanding how spatial scale influences the reliability of richness estimates from stacked ENMs.

While investigations of the relationship between richness estimates from stacked ENMs and spatial extent are scarce (Ortego & Knowles, 2020; Valencia‐Rodríguez et al., 2021), the effects of species’ biology on ENMs performance have been widely discussed in the literature (Castaño‐Quintero et al., 2020; Low et al., 2021; Velazco et al., 2017). In particular, studies agree that the model estimates of potential distributions are more accurate for species with narrow environmental limits than those for species with wider ones. Wide environmental limits are more difficult to estimate with ENMs due to the partial knowledge of species presence (i.e., occurrence points) and a small number of environmental variables used as model parameters (Cheng et al., 2021). For example, a study of 125 Neotropical plant species showed that it is more challenging to model accurately the distribution of species with large ranges (Velazco et al., 2017). This indicates that broad geographic ranges correspond to broad environmental conditions, although in some cases broad areas can be environmentally homogenous (e.g., deserts). At the same time, models may perform more poor for species with limited dispersal abilities (Della Rocca & Milanesi, 2020). Dispersal, a determinant of range size, may reduce a species’ ability to fully occupy its potential distribution as represented by the environmental limits or may result in ephemeral, sink populations outside its potential distribution (Scheele et al., 2017).

**FIGURE 1:**
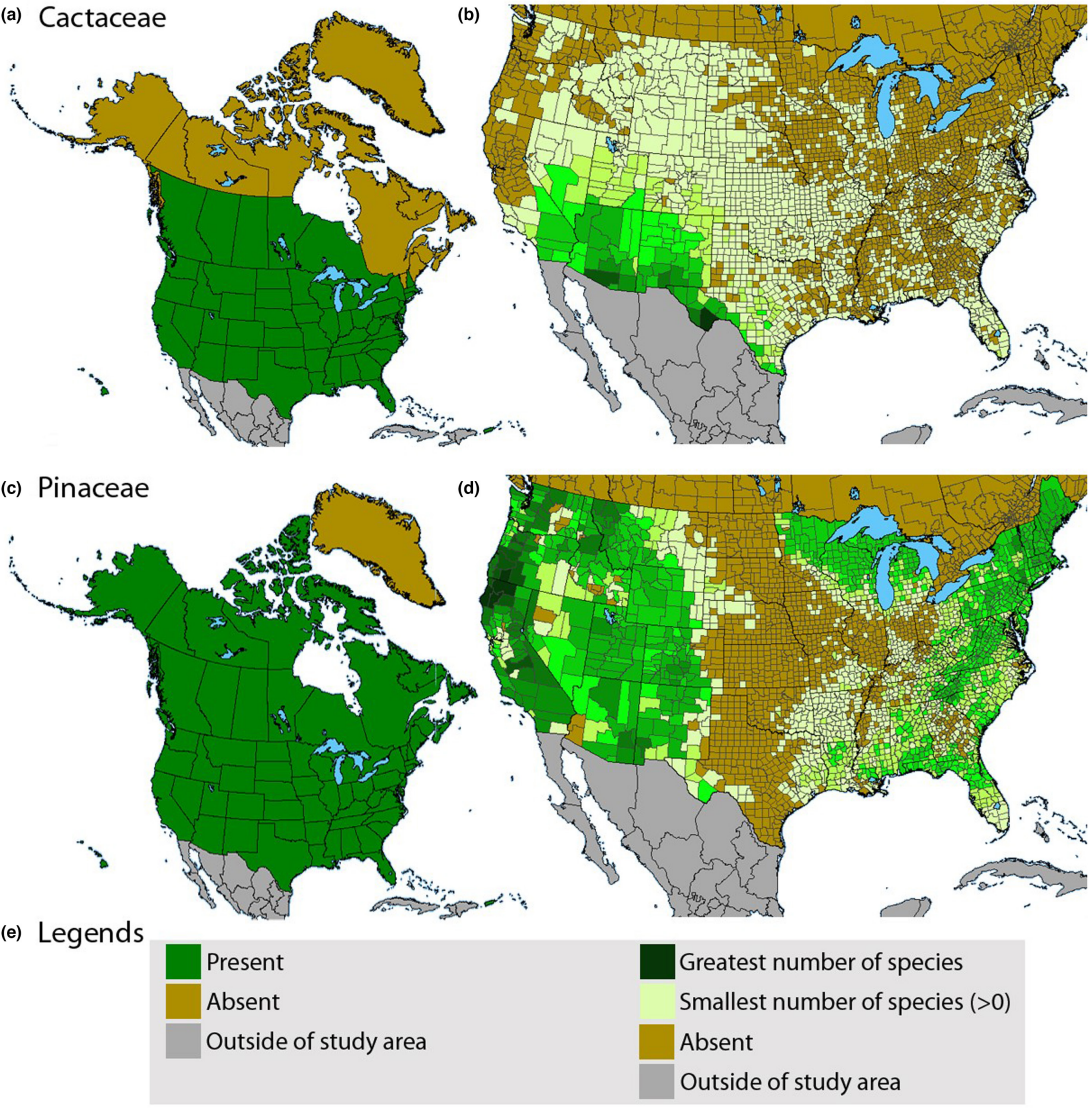
Geographic distributions of native Cactaceae and Pinaceae in North America north of Mexico. (a) Presence of Cactaceae in North America north of Mexico. (b) Heat map showing the relative number of species of Cactaceae in US counties. http://bonap.org/2015_SpecialtyMaps/Most%20Number%20of%20Native%20Species/Number%20of%20Species%20per%20Family%20Maps/rank%20024%20CACTACEAE.png. (c) Presence of Pinaceae in North America north of Mexico. (d) Heat map showing the relative number of species of Pinaceae in US counties. (e) Legend for maps showing state and province presence (left) and maps showing relative abundance of species (right). http://bonap.org/2015_SpecialtyMaps/Most%20Number%20of%20Native%20Species/Number%20of%20Species%20per%20Family%20Maps/rank%20054%20PINACEAE.png. Maps reproduced without modification from Biota of North America Program with written permission.

To improve our understanding of the effect of spatial extent on species’ richness estimated with stacked ENMs, we investigated Cactaceae and Pinaceae, two taxonomic groups with distinct biology and environmental requirements and limitations. We chose Cactaceae and Pinaceae families because they are relatively well understood taxonomically, appear prominently on the landscape, and are thus less likely to be missed during floristic surveys. We also selected them for their biological and ecological differences, evidenced by their life histories and distributions. The Pinaceae, or pine family, is the world's second‐most widely distributed conifer family (after Cupressaceae). While most of the species are found in temperate climates, they range from the subarctic to the tropics (Eckenwalder, 2009; Thieret, 1993). In contrast, Cactaceae has a native range limited to warm, arid regions of the Americas (Britton & Rose, 1963; Parfitt & Gibson, 2003). The global differences in distribution patterns of these families are also reflected within their North American ranges (Figure 1). Spatial extents ranged from 10^1^ to 10^7^ ha in our analyses.

Our objective was to assess the reliability of stacked ENMs as a predictors of species richness within differently sized geographic areas for the two taxonomic groups. We compared predictions of richness for Cactaceae and Pinaceae to floras of North America north of Mexico (i.e., the United States, Canada, and French territory of Saint‐Pierre and Miquelon; hereafter, North America). Based on the available, albeit limited, evidence for area size effect on richness estimates, we hypothesized that the correlation between richness from stacked ENMs and richness from published floras will increase with the spatial extent of the analysis. We also expected that this relationship would be stronger for Cactaceae because species richness for this family is concentrated in the southwestern USA and North Mexico, whereas Pinaceae richness covers large portions of western and eastern North America. Thus, the stacked ENMs would be less performant for Pinaceae than Cactaceae, given the higher environmental heterogeneity of the former.

## 2 MATERIALS AND METHODS

### 2.1 Sampling

#### 2.1.1 Sampling of floras

We sought to compare species richness estimated from stacked ENMs with richness from published species lists for 180 North American floras, which were vetted for minimum information standards (i.e., Palmer & Richardson, 2012) and accessioned by the Floras of North America project (http://botany.okstate.edu/floras/; also, in peer‐reviewed articles, e.g., Qian et al., 2007; Palmer, 2006; Table S1). We collected a stratified random sample of floras from the Floras of North America database. Our stratifications comprised six size classes in the flora from 10^1^ *< x ≤* 10^2^ ha to 10^6^ *< x ≤* 10^7^ ha (hereafter referred to by their upper bounds) within each of the Arctic, Pacific, or Atlantic drainage basins (Table S2), for a total of 18 strata. We used the stratifications to ensure a representative sampling of floras of different sizes from across North America. From each of the 18 stratifications, we randomly selected ten floras, for a total of 180 floras (Table S2). Specifically, we used randomized lists to choose within each stratification the first ten floras reasonably available (i.e., online or in hardcopy) and with species lists organized by family (Table S3). Details of all selected floras are given in Table S1.

#### 2.1.2 Pinaceae and Cactaceae species within floras

We selected Cactaceae because it is more of a specialist clade driven strongly by temperature, while Pinaceae is more of a generalist clade without strong climatic restrictions. We determined the species (if any) of Cactaceae and Pinaceae that occurred within each of the 180 North American floras selected (Table S3). We retained taxonomic information at the species level and did not record determinations of subspecies, varieties, or forms when present. Additional details regarding taxonomic incongruences and reconciliation are provided in Appendix S1.

#### 2.1.3 Occurrence data

After obtaining occurrence records from the Global Biodiversity Information Facility (GBIF, http://www.gbif.org/) for all reconciled names and their synonyms, we automated the selection of records using the “dismo” package (Hijmans et al., 2017) for R (Appendix S2).

We retained occurrence records based on three criteria, namely that the record was of a specimen or observation, could be readily georeferenced, and represented an individual occurring within its native geographic range. To retain records only of specimens and observations, we simply removed other kinds of records (e.g., fossil, literature, and unknown) from the initial download from GBIF. We used latitude and longitude coordinates obtained directly from the GBIF and, for the records without coordinates, we georeferenced (i.e., assigned coordinates) locality descriptions with GeoLocate (Rios & Bart, 2010). We determined the native geographic ranges of species to the level of state or province using Flora of North America (Parfitt & Gibson, 2003; Thieret, 1993) and to the level of the country using Conifers of the World: The Complete Reference (Eckenwalder, 2009) and Cactaceae: Descriptions and Illustrations of Plants of the Cactus Family (Britton & Rose, 1963). We removed all records that represented occurrences outside of these broadly defined native ranges. We also eliminated *Hylocereus undatus* (Haworth) Britton & Rose (dragon fruit, pitaya) from the Cactaceae dataset because it is extensively cultivated, invasive in some areas, and its native range is unknown (Britton & Rose, 1963; El Mokni et al., 2020).

#### 2.1.4 Environmental data

Our environmental data comprised the 19 bioclim variables from WorldClim ver.1 ((http://www.worldclim.org/) at a resolution of 2.5 arc‐min, elevation and soil data from the Food and Agriculture Organization of the United Nations (FAO, http://www.fao.org/), and climatic moisture index (Vörösmarty et al., 2005; available from http://databasin.org/), which is a ratio of annual precipitation to potential annual evaporation (Willmott & Feddema, 1992). The elevation dataset was derived from the Space Shuttle Radar Topography Mission at 30 m resolution (Fischer et al., 2008). Of the soil variables available within the Harmonized World Soil Database at 1 km resolution (FAO/IIASA/ISRIC/ISS‐CAS/JRC, 2012), we used those with very little missing data globally and no missing data for species’ occurrence records. Water capacity (AWC) and ten other soil variables representing top‐ and subsoil properties such as gravel, sand, clay, silt, and pH met our data quality requirements.

##### Generating Ecological Niche Models (ENMs)

We used Maxent 3.3.3 k (Elith et al., 2011; Phillips et al., 2004, 2006; Phillips & Dudík, 2008) to calibrate an ENM for each species with available occurrences and environmental variables. Maxent is a maximum entropy algorithm that requires only presence records and attempts to minimize overpredictions (Elith et al., 2011; Phillips et al., 2004, 2006; Phillips & Dudík, 2008). We used the default settings of Maxent 3.3.3 k, except that we applied a targeted background sampling to reduce the influence of sample selection bias (Phillips et al., 2009). The target groups were Pinaceae and Cactaceae species within Floras.

Initially, we used Maxent to generate ENMs for all species with nonzero sample sizes of occurrence records. We separated the records into 70% training and 30% testing subsets and calculated omission error (percentage of test presences incorrectly predicted absent or unsuitable) for each species model. We used the 10‐percentile training presence threshold to convert the predictions of continuous suitability to binary format (suitable and unsuitable classes). This threshold corresponds to the probability of environmental suitability associated with 10% training omission error (i.e., 10% of training presences incorrectly predicted as absent); areas with probability of suitability values below this threshold are reclassified as unsuitable and areas with probability above this threshold are reclassified as suitable.

Following the initial analyses, we divided species of Cactaceae and Pinaceae into three categories based on the number of occurrence records available for fitting ENMs: species with occurrences insufficient for modeling (≤6 occurrences), species with occurrences sufficient for model building only (i.e., training only; 7–99 occurrences), and species with occurrences sufficient for model building and testing (≥100 occurrences; 30% of records used for model testing). We determined the number of occurrences from the initial analysis in Maxent that identified records in null‐value regions of the mapped environmental variables (i.e., no data grid cells on coastlines and lakes), as well as records that were not spatially unique (i.e., falling in the same environmental grid cell). We generated models for the species with seven to 99 occurrence records by using all available records to train the models, thus leaving no records to independently test the model, but otherwise following the same protocols used for producing the initial models. We evaluated these models with training omission error instead of testing omission error. Only two species, both of Cactaceae, had insufficient records (i.e., ≤6 occurrences) and were excluded from additional modeling and downstream analyses: *Harrisia simpsonii* Small ex Britton & Rose and *Opuntia monacantha* Haw. We chose the minimum number of occurrences for species exclusion decisions, model building without testing, and modeling with 30% testing data based on prior studies (Papeş & Gaubert, 2007; Stockwell & Peterson, 2002; van Proosdij et al., 2016).

##### Processing and analyzing ENMs

We cropped the model output (potential distribution) for each species to the bounding box of each flora using a simple Python script written for use with the ArcGIS (ESRI, 2014) library (Appendix S2). We obtained a total of 22,680 cropped models representing 180 floras, 60 species of Pinaceae, and 66 species of Cactaceae. We used bounding boxes to account for edge effects; namely that disagreement between two models or a model and a flora is more likely at the edges of each than nearer to their centers and that disagreements at edges may be less meaningful than disagreements nearer to centers (Araujo & New, 2007; Power et al., 2001).

The bounding boxes were predictably larger than the floras, so we evaluated whether the differences between richness from stacked ENMs and richness from floras could be artifacts of the species–area relationship (Gleason, 1922: Gleason, 1925). We evaluated the effects of bounding boxes area (*A*) on species richness (*S*) using the *z* coefficient or slope of the linear species–area equation *S = cAz* when log‐transformed, log*S* = *z*log*A* + log*c*. To accomplish this, we modified the equation to calculate *z* for the sizes of the bounding boxes compared with the floras. Thus, our calculation of *z* = [[log(predicted *S*)−log(reported *S*) / [log(bounding box *A*) − log(reported *A*)]]]. We compared our *z* to values from the literature, usually within 0.1–0.35 in temperate and polar continental areas for organisms of all kinds (Morgan et al., 2011; Preston, 1960, 1962; Rosenzweig, 1995) and a slightly narrower range of 0.1–0.27 when calculated from the Floras database for vascular plants of North America north of Mexico (Qian et al., 2007). We expected that a *z* much larger than the reported *z* would indicate that the discrepancies between richness estimates from stacked ENMs and richness from floras were unlikely due to the sizes of the bounding boxes. We also visualized [log(predicted *S*)−log(reported *S*)] as a function of [log(bounding box *A*) – log(reported *A*)] using scatter plots.

To understand the reliability of the stacked ENMs as estimators of species richness, we examined differences between predicted versus reported species richness using linear models, and we measured the strength and directionality of the linear relationships using *R* ^2^ and slopes, respectively. We performed linear regressions for all floras and stacked ENMs and, independently, within each size class.

To explore the taxonomic biases in stacked ENMs predictions of richness relative to richness from floras, we used confusion matrix methods of sensitivity and specificity (presented in Table S4; reviewed in Zurell, Zimmermann, et al., 2020). Sensitivity is the proportion of species correctly predicted present (i.e., proxied by the prediction of suitable environments) out of all sampled species that occur in the flora (Peterson et al., 2011). Specificity is the number of species correctly predicted absent (i.e., proxied by the prediction of unsuitable environments) from a flora out of all sampled species that do not occur there (Peterson et al., 2011; Zurell, Zimmermann, et al., 2020). Notably, sensitivity cannot be calculated for floras with no reported presence (see equation in Table S4). We performed all statistical evaluations after removing unmodeled species of Pinaceae and Cactaceae from the lists obtained from the published floras.

## 3 RESULTS

### 3.1 Ecological niche models

We generated ENMs for 66 species of Cactaceae and 60 species of Pinaceae, for a total of 126 ENMs. We collated occurrence records sufficient for model building and testing (≥100) for 51 species of pines and 24 species of cacti (Table S3). Model testing showed that omission error decreased predictably (Hernandez et al., 2006; Reese et al., 2005) as the number of occurrence records increased (Figure S2). The average omission error for species of Cactaceae (18.96%) was higher than for species of Pinaceae (13.39%). The average omission error rates for both plant families were relatively low and within tolerable margins (5–20%) for models built from data records obtained from an online repository (Peterson et al., 2008). Notably, there were more species models in Cactaceae with omission error of >20% compared with models of species in Pinaceae.

### 3.2 Species richness

We found that *z* coefficients were highly variable but were larger for Cactaceae (1.06 ± 0.906 standard deviation) than Pinaceae (0.68 ± 1.05 standard deviation) on average. Our calculated *z* coefficients were considerably larger than the values reported in the literature, 0.1–0.35 in temperate areas (various organisms) and 0.1–0.27 from Floras database (vascular plants, North America north of Mexico). Thus, the differences in richness estimates from stacked ENMs and from floras were not artifacts of area differences between bounding boxes of ENMs and floras.

**FIGURE 2:**
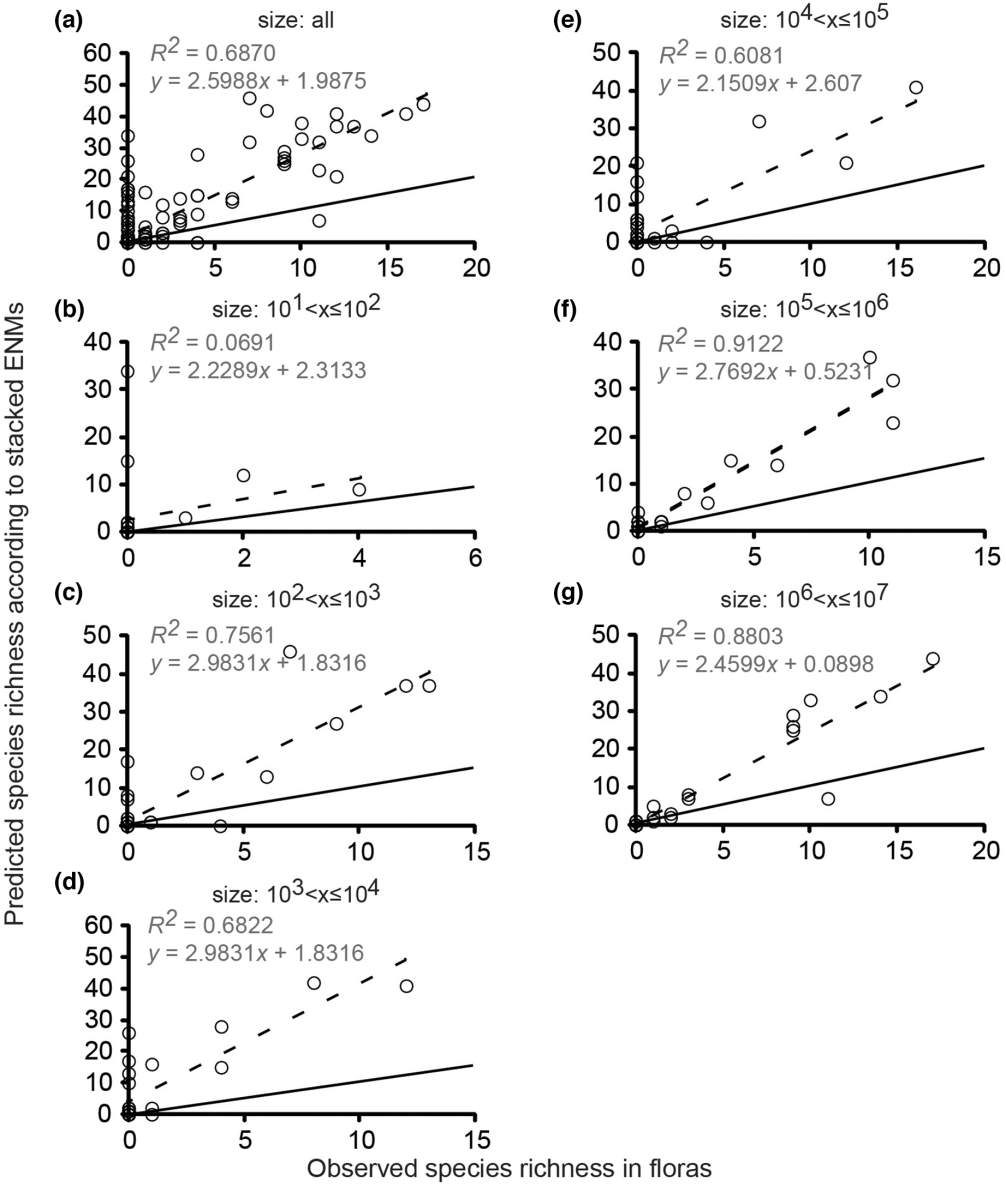
Relationships between reported and predicted species richness of Cactaceae from floras and stacked ENMs. The *R* ^2^ value and the equation for the regression model are displayed on each chart. Two lines are displayed on each chart; dashed line = regression line, solid line = line of equality. Individual graphs show the relationships between models and reported richness in (a) all floras, (b) size class 10^1^ *< x ≤* 10^2^ ha, (c) in size class 10^2^ *< x ≤* 10^3^ ha, (d) in size class 10^3^ *< x ≤* 10^4^ ha, (e) in size class 10^4^ *< x ≤* 10^5^ ha, (f) in size class 10^5^ *< x ≤* 10^6^ ha, (g) in size class 10^6^ *< x ≤* 10^7^ ha.

**FIGURE 3:**
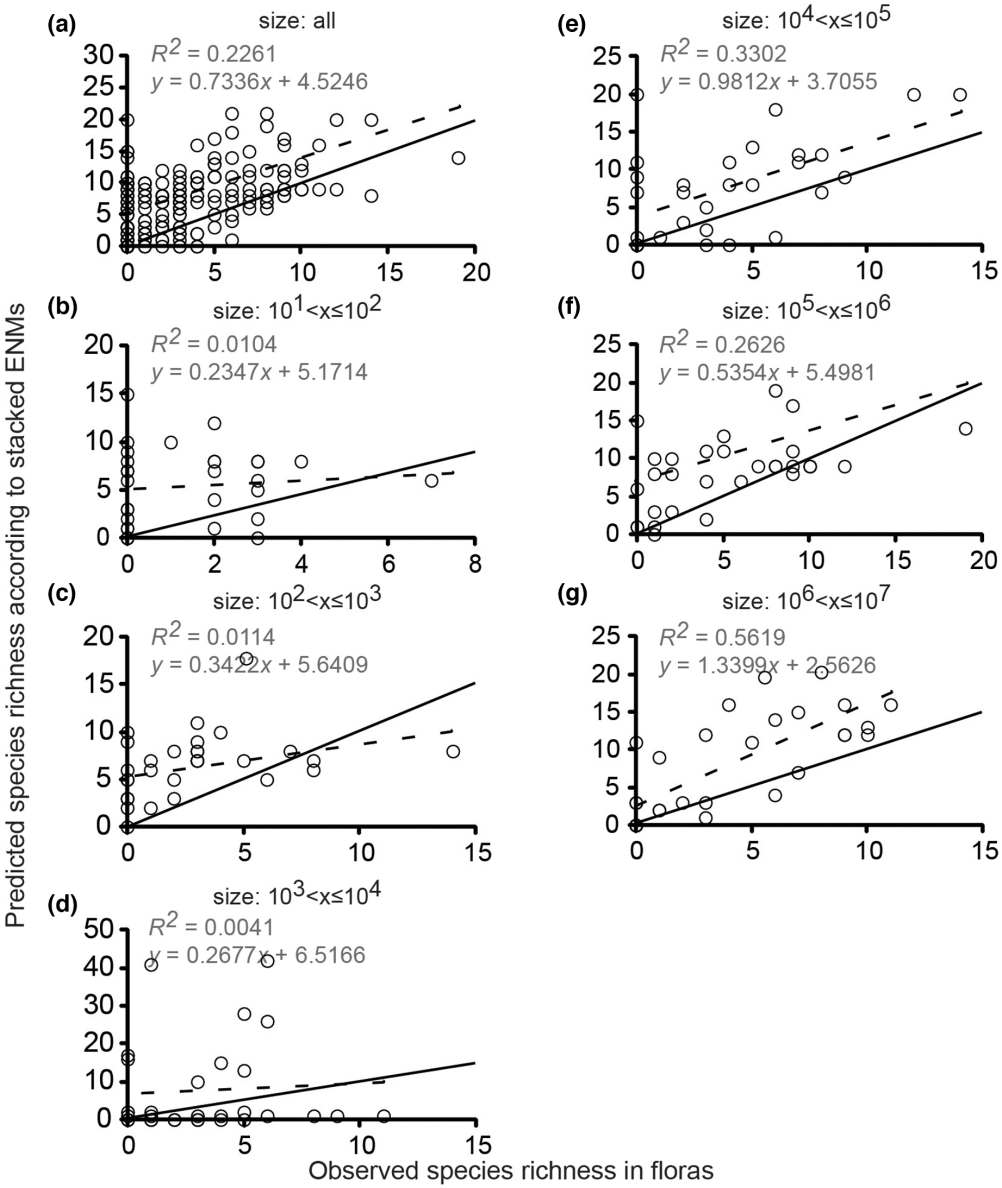
Relationships between reported and predicted species richness of Pinaceae from floras and stacked ENMs. The *R* ^2^ value and the equation for the regression model are displayed on each chart. Two lines are displayed on each chart; dashed line = regression line, solid line = line of equality. Individual graphs show the relationships between models and reported richness in (a) all floras, (b) size class 10^1^ *< x ≤* 10^2^ ha, (c) in size class 10^2^ *< x ≤* 10^3^ ha, (d) in size class 10^3^ *< x ≤* 10^4^ ha, (e) in size class 10^4^ *< x ≤* 10^5^ ha, (f) in size class 10^5^ *< x ≤* 10^6^ ha, (g) in size class 10^6^ *< x ≤* 10^7^ ha.

**FIGURE 4:**
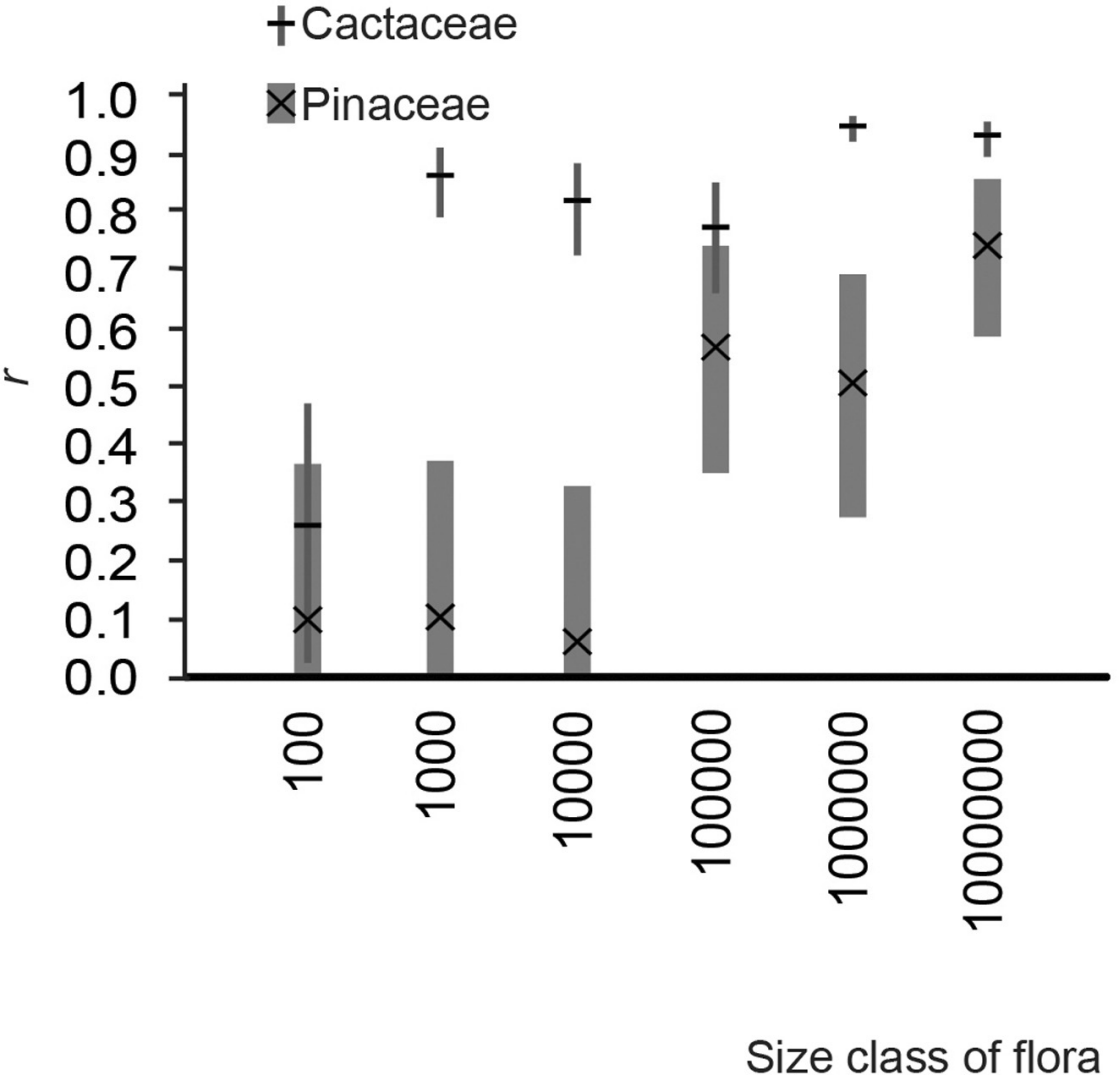
Trends in reported and predicted species richness by size class of floras. The *r* correlation coefficients (Fisher, 1915, 1921) for linear models of species richness reported in published floras and predicted by stacked ENMs. The *r* values are marked with horizontal lines for Cactaceae and “x” for Pinaceae. 95% confidence intervals for *r* are shown as black lines for Cactaceae and gray bars for Pinaceae. The confidence intervals are truncated at 0.

The slopes of regression lines showed a positive relationship between species richness reported in published floras and species richness predicted according to stacked ENMs for both Cactaceae (Figure 2, especially 2A) and Pinaceae (Figure 3, especially 3A). We detected positive relationships for floras in all six size classes (Figures 2b–g and 3b–g). In Cactaceae, the strength of the relationship between reported and predicted species richness generally increased across the spatial extents of floras, ranging from negligible, *R* ^2^ = 0.0691, in floras of 10^2^ ha to strong, *R* ^2^ = 0.9122 and *R* ^2^ = 0.8803, in floras of 10^6^ ha and 10^7^ ha, respectively (Figure 2). In Pinaceae, the strength of the relationship between reported and predicted richness also increased with spatial extent: from negligible, *R* ^2^ = 0.069, in floras of 10^2^ ha to strong, *R* ^2^ = 0.8803, in floras of 10^7^ ha. However, in Pinaceae, the trend was less gradual and continuous than in Cactaceae. Striking differences were evident between the smallest three size classes and the largest three (Figure 3). In Cactaceae, our regression analyses had correlation coefficients (*r*) with overlapping confidence intervals except for the extents of 10^2^ ha and 10^6^ ha (Figure 4). In Pinaceae, all confidence intervals of *r* overlapped (Figure 4).

**FIGURE 5:**
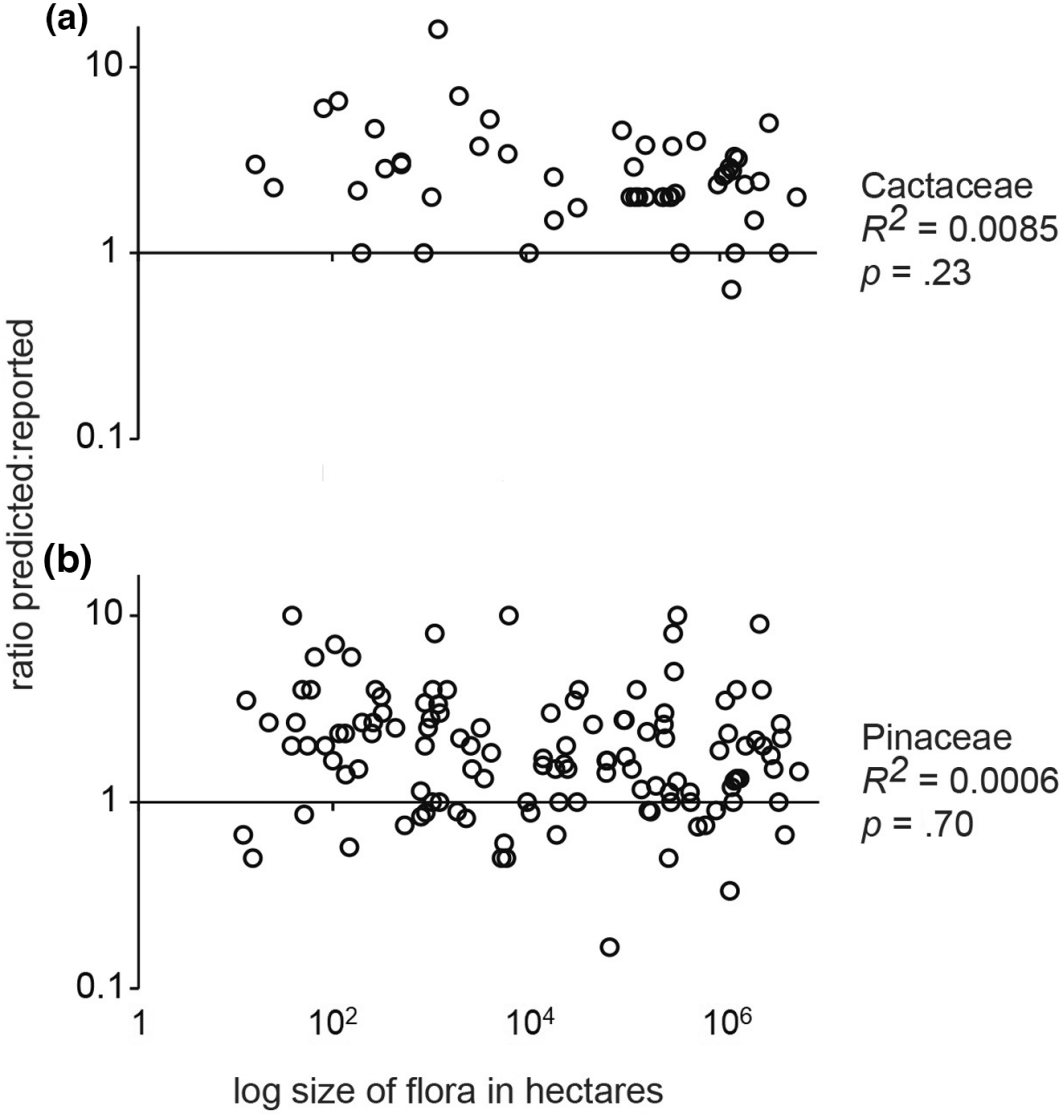
Size of flora area in hectares compared the ratio of species reported to species predicted by published floras and stacked models. Both axes shown in log scales, and *R* ^2^ values shown to the right of graphs. (a) Cactaceae and (b) Pinaceae.

Overall, the stacked ENMs showed low reliability and large biases in predictions of species richness. In Cactaceae, the average overprediction from stacked ENMs across all floras was 4.5 modeled species for every one reported species. Overprediction for cacti changed negligibly with spatial extent (Figure 5a; *R* ^*2*^ = 0.0085). Pinaceae also showed little change in overprediction with increasing extent (Figrue 5b; *R* ^*2*^ = 0.0006). In Pinaceae, the average overprediction was 3.6 modeled species for every reported. Underpredictions were more common for pines than cacti (Figure 5a,b). The trends in overprediction by stacked ENMs could not be entirely attributed to a species–area effect caused by the bounding boxes being larger than the floras (Figure S1; Table S3). Across all size classes, Cactaceae showed less frequent incorrect predictions of richness >0 when no cactus species were present (Figure 2, *y*‐intercepts) compared with the same case in Pinaceae (Figure 3, *y*‐intercepts). Still, neither family showed a clear relationship between flora size and reliability of stacked ENMs predictions for the situations of no species presence records in floras. Trends in biases toward overprediction and underprediction were visually apparent using the lines of equality (Figures 2 and 3, solid lines).

### 3.3 Specificity, sensitivity, and similarity

**FIGURE 6:**
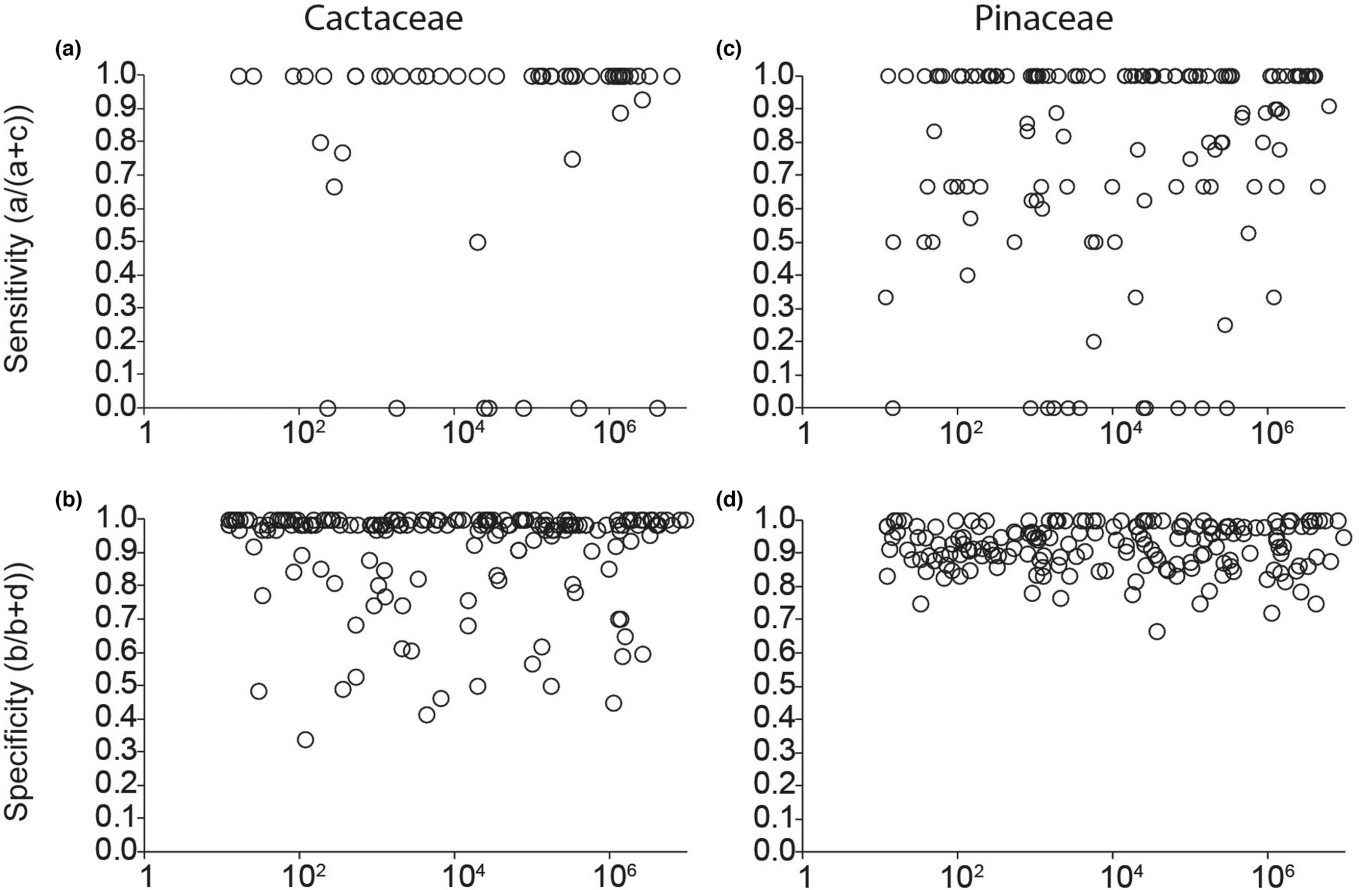
Sensitivity and specificity index metrics. Note that all graphs have a logarithmic x‐axis and that sensitivity was calculated only for those floras with reported presences (i.e., nonzero denominator for the metric, see Table S4). (a) Sensitivity versus size in hectares in Cactaceae. (b) Specificity versus size in hectares for Cactaceae. (c) Sensitivity versus size in hectares in Pinaceae. (d) Specificity versus size in hectares for Pinaceae. For all subfigures, refer to values derived from the confusion matrix (Table S4).

The stacked ENMs for Cactaceae showed high sensitivity within the bounding boxes of most floras, indicating the reliability of predicting the number of species reported in floras (Figure 6). Consequently, this means that taxon‐specific omission rates for the floras were low. In contrast, models of Pinaceae more frequently omitted species from floras in which they were reported to occur (Figure 6). The results for specificity showed the stacked ENMs of Pinaceae generally outperformed those of Cactaceae across all floras (Figure 6). This means that Pinaceae species were less often predicted present when they were unreported (i.e., fewer commission errors) than for Cactaceae. The sensitivity and specificity metrics did not appear to correlate with spatial extent for either plant family (Figure 6).

## 4 DISCUSSION

In this study, we evaluated species richness estimates from staked ENMs against richness from floras, at various geographic extents (10^1^–10^7^ ha), for Pinaceae and Cactaceae. We found that stacked ENMs were poor predictors of species richness, except at large geographic extents. Richness of Cactaceae was generally overpredicted compared with richness from floras of all sizes, indicated by high sensitivity and low specificity values, whereas reliability of Pinaceae richness estimates was more variable (richness over and underpredicted). Stacked ENMs representing larger floras with greater extents had observable but not significantly more accurate richness estimates for Cactaceae. Our results (*r* and *R* ^*2*^ values) show stronger, positive relationships between reported and estimated species richness for Cactaceae than Pinaceae at all extents, especially for floras of 10^3^ and 10^4^ ha.

The high sensitivity of cacti richness estimates could indicate a sampling bias; that is, published floras represent areas that are well‐explored and well‐represented among occurrence records and, therefore, are readily predicted as possessing suitable environments for the modeled species (Araújo & Guisan, 2006). However, if high sensitivity in cacti were related to sampling biases correlated with published floras, we would expect to obtain similarly high levels of sensitivity for Pinaceae, and we do not. An alternative explanation is that the omission error calculated for models of each species within Maxent is too stringent by considering only whether or not an occurrence point overlaps with a pixel that is predicted to have a suitable environment. Notably, our study's pixels represented ~100 ha (i.e., 30 arc seconds or ~1 km). Less stringent comparisons, such as those that consider neighboring pixels and pixel position within models (e.g., center or edge), may be more informative for spatial evaluation of models, and these comparisons are correlated with evaluation metrics frequently used for ENMs (Sarquis et al., 2018). Thus, omission error may be reduced at the coarser scale of the bounding boxes of floras, which represented <100 ha at minimum (Table S2). However, our results for Pinaceae demonstrate that omission error rates of individual species’ models may fall within acceptable range of values (Grant & Kalisz, 2020) and may be higher at the coarser scale of the bounding boxes (Figure 5c; Appendix S2). Ultimately, Cactaceae and Pinaceae may differ in the error rates of their ENMs and consequently stacked ENMs for biological reasons (e.g., dispersal abilities and biotic interactions), which could be further explored in subsequent studies.

We expected that stacked ENMs would show biases toward overprediction of species richness because they are built from ENMs, which estimate suitable environments and do not consider limitations to ranges caused by biotic interactions, geographic barriers, or other unknown, unmeasured factors (Cheng et al., 2021; Mantovano et al., 2021; Sillero & Barbosa, 2021; You et al., 2018). Nevertheless, previous authors have asserted that stacked ENMs may be useful in predicting diversity patterns if not quantifying them exactly (Boavida‐Portugal et al., 2022). Cactaceae showed consistent overprediction of richness at all spatial extents, while Pinaceae richness was both over and underpredicted at all extents (Figures 2 and 3, 5).

**FIGURE 7:**
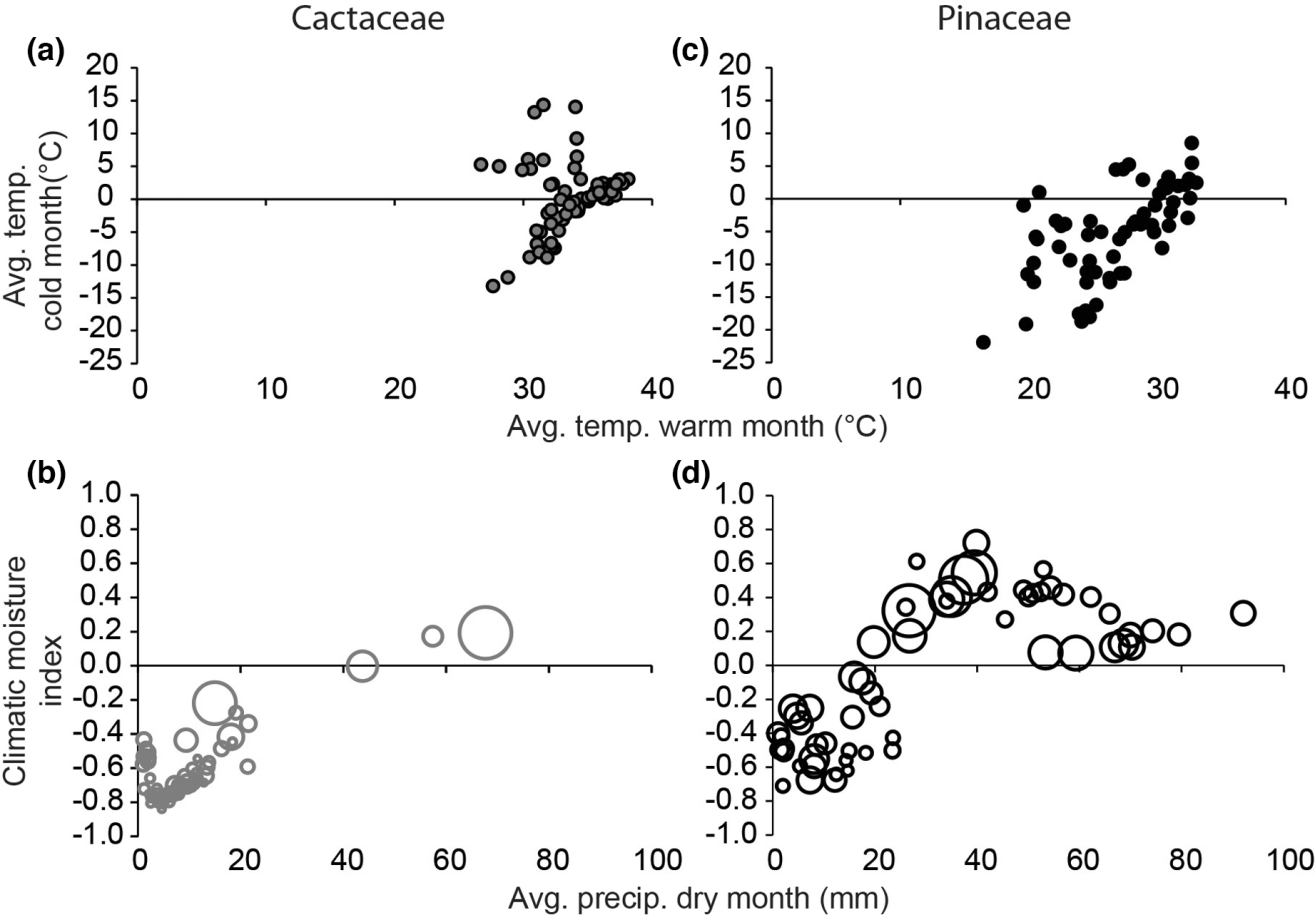
Environmental breadth of Cactaceae and Pinaceae according to selected climatic variables. Averages for locations of occurrence data points for each species. (a) Environmental breath of Cactaceae along temperature extreme axes, warmest and coldest months. (b) Environmental breath of Cactaceae along precipitation axes, driest month, and climatic moisture index. Relative bubble size compares precipitation during the wettest month. (c) Environmental breath of Pinaceae along temperature extreme axes, warmest and coldest months. (d) Environmental breath of Pinaceae along precipitation axes, driest month, and climatic moisture index. Relative bubble size compares precipitation during the wettest month.

The richness estimates from stacked ENMs had higher sensitivity for cacti than for pines, whereas specificity was higher for pines than cacti (Figure 6). These metrics indicate that stacked ENMs more accurately estimated Cactaceae species richness for floras that contained Cactaceae species than for floras that did not list cacti species. Cacti have more strict limitation of temperature and precipitation compared with pines (Figure 7), and these variables likely contributed to better estimations of environmental conditions associated with presence for cacti than for pines. The low specificity of richness estimates for cacti may be due to the limited dispersal ability of cacti and thus their inability to fully occupy their environmentally suitable range (Bregman, 1988). Some species lack known dispersal mechanisms, while others may have limited dispersal due to animal dependence (Guerrero et al., 2012). On the contrary, for pines, temperature and precipitation predictors within the ENMs may have been sufficient to improve specificity (correctly predicting the absence of pines). However, overall, unmeasured variables such as edaphic or biotic features may be the primary drivers of pines’ distributions and thus our ENMs had limited ability to estimate suitable conditions for pines; hence, the lower sensitivity of richness estimates compared with that for cacti (Dobrowski et al., 2011; McPherson & Jetz, 2007; Pöyry et al., 2008; Syphard & Franklin, 2010). Integrating edaphic and biotic variables is critical for ENMs and other similar applications for some taxonomic groups (Velazco et al., 2017; Wisz et al., 2013).

In this study, we did not quantify and compare the environmental limits of Pinaceae and Cactaceae, but, in general, the pines seem to occupy a wider range of environments than the cacti, at least by some measures (Figure 6; and as follows). The global distributional range of cacti spans from about 50°N latitude to the equator, whereas pines are found from 70°N to just south of the equator (Eckenwalder, 2009; Flora of North America Editorial Committee, 1993). The broader distributional range of Pinaceae is likely representative of the species’ broad environmental tolerances, from polar to tropical and rainforest to desert regions (Eckenwalder, 2009). The genus *Pinus* L. is thought to have one of the widest ecological ranges among woody plants (Eckenwalder, 2009). On the contrary, Cactaceae have xerophytic to mesic tolerances and generally do not occur in polar or very wet areas (Figure 7; Parfitt & Gibson, 2003). A study showed that the species richness of several cactus genera declines sharply outside of a very narrow annual temperature range (Cody, 1991). The narrower environmental breadth of Cactaceae may be more reliably estimated with ENM than the wider environmental breadth of Pinaceae. However, the environmental breadths of species should drive stacked model reliability, and these cannot necessarily be inferred from the environmental breadth of the family.

Cactaceae and Pinaceae may also differ in the importance of abiotic variables as drivers of their broad‐extent geographic ranges (Benavidez et al., 2018; Ding et al., 2021). It is well documented that abiotic variables play a role in determining the geographic range of many plant and animal species, especially at large spatial extents (Leach et al., 2016). Fewer studies documented biotic interactions as determinants of geographic ranges at large extents (Sheth et al., 2020). The geographic distributions of Pinaceae are likely profoundly shaped by historical and ongoing competition with flowering plants (Ding et al., 2021; Ramos‐Dorantes et al., 2017) and the availability of fungal symbionts (Mestre et al., 2020; Steidinger et al., 2020). In contrast, for Cactaceae environmental aridity is expected to have been the driving force in geographic radiations (Aquino et al., 2021).

Additionally, the probable importance of biological interactions at small geographic extents may help explain the poor performance of stacked ENMs for the floras in the smaller classes (Figures 2, 3, 4). Previous authors have speculated that stacked ENMs may more reliably predict the species richness of most organisms if abiotic and biotic variables can be integrated into the models (Feng et al., 2019; Johnson et al., 2019). Biotic variables are important even at broad geographic scales, though we are suggesting here that this may be taxon‐specific, that is, less important for cacti and more important for pines.

Studies employing stacked ENMs to infer species richness face several challenges, generally divided into pre‐ and postmodeling challenges. Premodeling challenges refer primarily to the limitations of data availability. In our study, the size of occurrence datasets available for ENMs had a stronger effect on model performance of Pinaceae than Cactaceae. Models trained with larger presence datasets tend to perform better due to improved sampling of environmental tolerances of species and reduced sampling bias (Araújo & Guisan, 2006; Stockwell & Peterson, 2002). Occurrence data are rapidly being digitized and disseminated online (Reginato & Michelangeli, 2020; Zurell, Franklin, et al., 2020; Petersen et al., 2021), but the records are available for a small percentage of existing specimens, which may themselves represent a fraction of global plant diversity (Marsico et al., 2020). Additionally, collections are often reduced to plants that are easy to acquire due to their accessible locations (Elith & Leathwick, 2009; Kadmon et al., 2004) whereas factors such as difficulty of handling and preserving cacti specimens (Baker et al., 1985; Fosberg, 1932) or narrow endemism and rarity (Ferrier & Guisan, 2006; Papeş & Gaubert, 2007) may limit collecting efforts. In our study, we eliminated one pine species and eleven cactus species (representing the complete elimination of four genera of Cactaceae) due to limited or no occurrence records. Premodeling challenges may also include issues related to data reliability and taxonomic reconciliation (Franz & Peet, 2009; Holt, 2009; Sarkar, 2007). The latter was fairly straightforward in our case but could be prohibitive for some taxonomic groups. Postmodeling challenges pertain to data interpretation, especially in the absence of a reference such as a published flora or surveyed vegetation plot. Caution must be exercised in making strong inferences based on stacked ENMs because of their potential for commission (type I) and omission (type II) errors and the confounding effects of unmodeled parameters, such as biotic interactions or other environmental variables. Our results show that stacked ENMs applied to small geographic extents may require particularly cautious interpretation, although the effect of geographic extent may be somewhat taxon‐dependent (Figure S1). We believe that our results provide new insights into the importance of considering species’ unique biology when using stacked ENMs to estimate species richness and help further elucidate the effects of geographic scale.

Other methods used to estimate species co‐occurrences include joint species distribution models (JSDMs) that consider the covariance between species occurrences (Pollock et al., 2014) and spatially explicit species assemblage modeling (SESAM) that incorporates macroecological constraints and assembly rules (Guisan & Rahbek, 2011). Species richness estimates obtained with JSDMs and SESAM are comparable to those obtained with stacked ENMs, as evidenced by recent comprehensive studies. For example, a study contrasting species richness estimates from stacked ENMs and JSDMs for bird and tree species found no significant differences or improvements for small subsets of taxa, such as rare species, and the overestimation of species richness was higher for JSDMs than stacked ENMs (Zurell, Zimmermann, et al., 2020). The similarity in richness estimates from stacked ENMs and JSDMs was also supported by a broader taxonomic study that included herbaceous plants, trees, butterflies, and birds and considered various possible methodological effects such as modeling algorithm, evaluation metric, interactions among environmental variables, and model parameter uncertainty (Norberg et al., 2019). Richness of Mediterranean bird communities was more accurately estimated with stacked ENMs than SESAM (Di Febbraro et al., 2018); however, a simplified SESAM implementation applied to plant communities in the Swiss Alps reduced the overprediction of species richness (D'Amen, Dubuis, et al., 2015).

The results of our study show that stacked ENMs are relatively poor predictors of species richness and specific taxonomic composition for two families of vascular plants, Cactaceae and Pinaceae, at all spatial extents studied, namely 10^1^–10^7^. Thus, stacked ENMs should be used with caution to estimate biodiversity. The tendency of the stacked ENMs toward overprediction may help to place a tentative upper bound on the richness of some taxa (e.g., Cactaceae). However, the degree or even occurrence of overprediction may vary by taxon, and taxonomic effects on stacked ENMs may be difficult to determine *a priori*.

## AUTHOR CONTRIBUTIONS

**Mir Muhammad Nizamani:** Formal analysis (equal); software (equal); writing – original draft (equal); writing – review and editing (equal). **Monica Papes:** Conceptualization (equal); methodology (equal); project administration (equal); resources (equal); software (equal); supervision (equal); writing – review and editing (equal). **AJ Harris:** Conceptualization (equal); data curation (equal); formal analysis (equal); investigation (equal); methodology (equal); project administration (equal); resources (equal); software (equal); supervision (equal); validation (equal); visualization (equal); writing – original draft (equal); writing – review and editing (equal). **Hua‐Feng Wang:** Project administration (equal); supervision (equal); writing – review and editing (equal).

## FUNDING INFORMATION

This study was supported by the National Natural Science Foundation of China (32160273), the Project of Sanya Yazhou Bay Science and Technology City (SCKJ‐JYRC‐2022‐83), and an open funding from Huadong Normal University (SHUES2021A08 and SHUES2022A06).

## CONFLICT OF INTEREST STATEMENT

The authors declare no conflicts of interest.

## Supporting information


Appendix 1:

Appendix 2:

Figure S1:

Figure S2:

Table S1:

Table S2:

Table S3:

Table S4:

## Data Availability

Datasets and programming codes are available in Supporting Information.
