# Supplementary material for: How does spatial extent and environmental limits affect the accuracy of species richness estimates from ecological niche models? A case study with North American Pinaceae and Cactaceae
Source: Ecol Evol. 2023 Apr 21;13(4):e10007. doi: 10.1002/ece3.10007 (PMC10121319; doi:10.1002/ece3.10007)
Supplement: Supplementary file 1 — Appendix 1: [file ECE3-13-e10007-s001.docx]

**Appendix 1:** Supporting information regarding methods

We sought to ensure that there was no taxonomic incongruence at the family-level among the 180 floras on the circumscription of Cactaceae and Pinaceae. For the cacti, avoiding incongruence was fairly straightforward. Species of Cactaceae have long been easily recognized for their distinctive succulent morphology. The circumscription of Cactaceae has been stable since prior to the turn of the 20th century (Parfitt & Gibson, 2003; Hernández‐Hernández et al., 2011), and is supported by more recent molecular evidence such an inversion within the chloroplast genome shared among cactus species (i.e., a synapomorphy) (Parfitt & Gibson, 2003; Britton & Rose, 1963; Hernández‐Hernández et al., 2011). Thus, for pines, the situation was more complex, because as late as the 1950s Pinaceae included many genera that are now circumscribed among distantly related families (Eckenwalder, 2009). Therefore, we treated Pinaceae as inclusive of 11 genera following Eckenwalder (Eckenwalder, 2009): *Abies* Mill., *Cathaya* Chun & Kuang, *Cedrus* Trew, *Keteleeria* Carrière, *Nothotsuga* H.H. Hu ex C.N. Page, *Pseudolarix* Gordon, *Picea* A. Dietr., *Pinus* L., *Pseudotsuga* Carrière, *Larix* Mill., and *Tsuga* (Endl.) Carrière. From each flora, we recorded only species from these 11 genera as Pinaceae, regardless of the more inclusive treatments encountered within some floras.

For all species recorded from among the 180 floras, we reconciled taxonomy. Taxonomic reconciliation was important so that the species richness of each flora was not biased by differences of taxonomic opinion among authors of the floras. For Pinaceae, we used the nomenclature in Flora of North America North of Mexico. Vol. 2 Pteridophytes and Gymnosperms (Thieret, 1993), except for non-native species, for which we used Conifers of the World: The Complete Reference (Eckenwalder, 2009). For Cactaceae, we followed Flora of North America North of Mexico. Vol. 4 Magnoliophyta: Caryophyllidae, Part 1 (Thieret, 1993) except for one questionably native species, which reconciled to *Rhipsalis baccifera* (Sol.) Stearn according to the Tropicos database (http://www.tropicos.org/), and a recently described species *Echinocereus yavapaiensis* M.A. Baker, for which we followed the author of the species prologue (Baker et al., 1985). We used the same sources to generate lists of synonyms for each reconciled name. Following reconciliation, our 180 floras contained, in total, 78 species of Cactaceae and 61 species of Pinaceae.

Our script returned no records for nine species of Cactaceae (Consolea corallicola Small, Harrisia aboriginum Small ex Britton & Rose, Opuntia cubensis Britton & Rose, Pereskia aculeata Mill., Cylindropuntia kelvinensis (Grant & Grant) P.V.Heath, Opuntia triacantha (Willd.) Sweet, Opuntia stricta (Haw.) Haw., Pediocactus winkleri K.D. Heil, Pilosocereus robinii (Lem.) Byles & G.D. Rowley, and Sclerocactus whipplei (Engelm. & J.M. Bigelow) Britton & Rose and one species of Pinaceae (Pinus cedrus L.) because there were none present in GBIF.
