# Supplementary material for: How does spatial extent and environmental limits affect the accuracy of species richness estimates from ecological niche models? A case study with North American Pinaceae and Cactaceae
Source: Ecol Evol. 2023 Apr 21;13(4):e10007. doi: 10.1002/ece3.10007 (PMC10121319; doi:10.1002/ece3.10007)
Supplement: Supplementary file 2 — Appendix 2: [file ECE3-13-e10007-s003.docx]

**Appendix 2:** Computer scripts used in this study

#R script for downloading from GBIF with instructions as comments (#)

#Before you begin, be sure to download the R language and the dismo package

#Use the R GUI to navigate to the working directory of your csv file containing taxon names

#You can paste this script into the GUI. If you get an error, paste line by line.

data <- read.csv("test_array.csv", header=F)$V2 #where "test_array.csv" is the name of your data file and $V# is the list of species within test_array.csv

species_list <- as.character(data)

species_list <- do.call(rbind, strsplit(species_list, ' '))

species_list[,2] <- paste(species_list[,2], '*', sep='')

for (i in 1:nrow(species_list)) {

results <- gbif(species_list[i,1], species_list[i,2], download=TRUE, concept=FALSE, geo=FALSE, removeZeros=FALSE, ntries=50, nrecs=1000) #change species_list[i,2] to "*" (with the quotes) if you are downloading all species within a genus

#In the line above, you can obtain synonyms for your taxa using GBIF taxonomy by changing concept=FALSE to concept=TRUE

write.table(results, file = "DATA.csv", append=TRUE, sep=",")

#The results will be saved in the current working directory to a file called DATA.csv

}

#Python script for clipping models to bounding boxes with comments (#)

#Note: you may need to make changes depending on where and how you store your files and how you want/need your output to be named and stored.

import arcpy

from arcpy import env

env.workspace = "D:/your workspace"

import os

from os import *

chdir("D:/your workspace")

directory = getcwd()

maindir = listdir(directory)

pointdata = file("points for your bounding boxes in CSV format.csv", "r").readlines() #rows contain unique identifiers for each box, columns are the points defining the box. See below

for i, line in enumerate(pointdata):

if i > 0:

getpoints = line.split(",")

west = float(getpoints[5]) #change 5 to the number of the column containing the western boundaries MINUS 1

east = float(getpoints[6]) #change 6 to the number of the column containing the eastern boundaries MINUS 1

north = float(getpoints[3]) #change 3 to the number of the column containing the northern boundaries MINUS 1

south = float(getpoints[4]) #change 4 to the number of the column containing the southern boundaries MINUS 1

boxname = getpoints[0]

polypoints = [arcpy.Point(west, north), arcpy.Point(west, south), arcpy.Point(east, north), arcpy.Point(east, south)]

for f in maindir:

if ".asc" in f: #Will crop or attempt to crop all asciis in the "your workspace" directory. So, remove any .asc files that should not be cropped.

inputname = directory + "/" + f

inputcoords = str(east) + " " + str(south) + " " + str(west) + " " + str(north) # observe correct order of points

output = directory + "/" + f + "/" + boxname

Outfile = arcpy.Clip_management(inputname, inputcoords, output, "#", "#", "NONE", "MAINTAIN_EXTENT")
