# Supplementary figures and images for: How does spatial extent and environmental limits affect the accuracy of species richness estimates from ecological niche models? A case study with North American Pinaceae and Cactaceae
Source: Ecol Evol. 2023 Apr 21;13(4):e10007. doi: 10.1002/ece3.10007 (PMC10121319; doi:10.1002/ece3.10007)

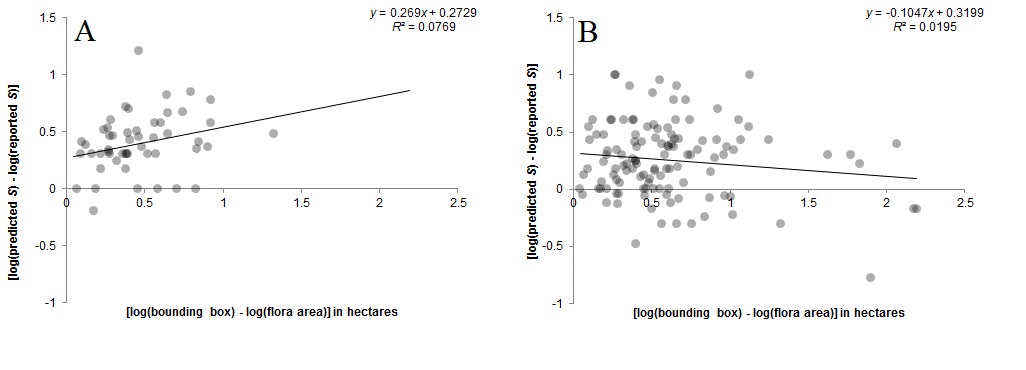

Supplement: Supplementary file 3 — Figure S1: [file ECE3-13-e10007-s006.jpg]

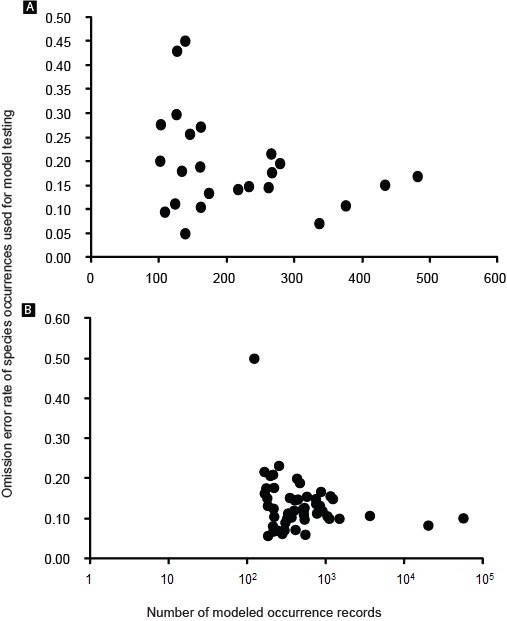

Supplement: Supplementary file 4 — Figure S2: [file ECE3-13-e10007-s004.jpg]
