# Supplementary material for: How does spatial extent and environmental limits affect the accuracy of species richness estimates from ecological niche models? A case study with North American Pinaceae and Cactaceae
Source: Ecol Evol. 2023 Apr 21;13(4):e10007. doi: 10.1002/ece3.10007 (PMC10121319; doi:10.1002/ece3.10007)
Supplement: Supplementary file 5 — Table S1: [file ECE3-13-e10007-s008.docx]

**Table S1:** **The spatial extents of bounding boxes**

| **Flora Accession Number** | **Drainage Basin** | **Size Class** | **Site Name** | **Actual Size in ha** | **Bounding Box in ha** | **Log(10) Actual Size** | **Log(10) Bounding Box Size** | **Absolute value of size difference between flora and bounding box** |
| --- | --- | --- | --- | --- | --- | --- | --- | --- |
| 48 | Pac | 10000000 | Nevada Test Site Ash Meadows and Nellis Air Force Base | 1424494 | 2490736 | 6 | 6 | 1066242 |
| 58 | Pac | 1000000 | Cache and Rich County | 571095 | 1103965 | 6 | 6 | 532870 |
| 118 | Pac | 1000 | Tumamoc Hill | 352 | 1022.19 | 3 | 3 | 670.1898 |
| 434 | Pac | 10000000 | Vancouver and Queen Charlotte Islands | 4060861 | 27312571 | 7 | 7 | 23251710 |
| 587 | Pac | 1000000 | Toiyabe Mountains | 323733 | 2721069 | 6 | 6 | 2397336 |
| 600 | Pac | 10000 | Chiricahua National Monument | 4247.6 | 10221.9 | 4 | 4 | 5974.298 |
| 707 | Atl | 1000000 | Houston County | 149184 | 227096.5 | 5 | 5 | 77912.51 |
| 725 | Pac | 10000 | Rosemont Area | 6475 | 11925.55 | 4 | 4 | 5450.548 |
| 811 | Pac | 10000000 | Aleutian Islands | 1766639 | 2878000 | 6 | 6 | 1111361 |
| 868 | Pac | 1000000 | Adak Island | 71118 | 141100 | 5 | 5 | 69982 |
| 921 | Arc | 10000000 | Devon Island | 5305356 | 19152856 | 7 | 7 | 13847500 |
| 1021 | Pac | 10000000 | San Diego County | 1089090 | 1375356 | 6 | 6 | 286266.4 |
| 1032 | Pac | 100 | Richvale vernal pools | 32.4 | 170.365 | 2 | 2 | 137.965 |
| 1047 | Arc | 10000000 | South Central North Dakota | 1257186 | 2816644 | 6 | 6 | 1559458 |
| 1072 | Arc | 10000000 | Wood Buffalo Park | 4480700 | 14218660 | 7 | 7 | 9737960 |
| 1132 | Arc | 100000 | Mortlach District | 76146 | 155798.8 | 5 | 5 | 79652.77 |
| 1278.2 | Atl | 10000 | Chandelier Islands | 4300 | 6900 | 4 | 4 | 2600 |
| 1476 | Atl | 10000 | Chimney Rock Area | 3300 | 12181.1 | 4 | 4 | 8881.095 |
| 1522 | Pac | 100000 | Crater Lake National Park | 64884.68 | 143106.6 | 5 | 5 | 78221.89 |
| 1870 | Pac | 100000 | Crater Lake National Park | 64884.68 | 143106.6 | 5 | 5 | 78221.89 |
| 2022 | Arc | 10000000 | Melville Hills region | 3300000 | 7626899 | 7 | 7 | 4326899 |
| 2116 | Arc | 10000000 | Southampton Island | 3421295 | 9046210 | 7 | 7 | 5624915 |
| 2164 | Arc | 1000000 | Barnes County | 391865.2 | 605136.4 | 6 | 6 | 213271.2 |
| 4750 | Pac | 1000000 | Nevada test site | 350000 | 651134.9 | 6 | 6 | 301134.9 |
| 4778 | Arc | 100000 | Salisbury Island | 80440 | 212700 | 5 | 5 | 132260 |
| 4783 | Arc | 100000 | Winter Island | 80440 | 212700 | 5 | 5 | 132260 |
| 4979 | Pac | 1000000 | Driftwood Valley Region | 12466 | 26576.94 | 4 | 4 | 14110.94 |
| 5208 | Arc | 10000000 | Northern Ellesmere Island | 103600 | 260062.1 | 5 | 5 | 156462.1 |
| 10042 | Atl | 1000 | Seven Mile Beach | 348612 | 946462.6 | 6 | 6 | 597850.6 |
| 10603 | Atl | 1000 | Raven Rock State Park | 1316232 | 1974019 | 6 | 6 | 657786.9 |
| 10834 | Atl | 100000 | Delaware County | 1435890 | 1685847 | 6 | 6 | 249956.1 |
| 11041 | Atl | 10000000 | New Jersey | 47656 | 130840.3 | 5 | 5 | 83184.3 |
| 11259 | Arc | 100000 | Montreal Islands | 1064 | 2555.475 | 3 | 3 | 1491.475 |
| 11295 | Atl | 10000000 | Maryland | 49919 | 171216.8 | 5 | 5 | 121297.8 |
| 11316 | Atl | 100000 | Detroit | 3213300 | 8085947 | 7 | 7 | 4872647 |
| 11317 | Atl | 1000 | Parkedale Farm | 37020 | 73938.4 | 5 | 5 | 36918.4 |
| 11575 | Arc | 1000 | Laval University Campus | 32640 | 53664.97 | 5 | 5 | 21024.97 |
| 11586.002 | Arc | 1000000 | East side of Algonquin Provincial Park | 473060 | 1435155 | 6 | 6 | 962094.5 |
| 11663 | Arc | 1000 | Cataraqui Marsh area | 3600 | 6559.051 | 4 | 4 | 2959.051 |
| 11723 | Arc | 1000 | Royal Botanical Gardens | 680132 | 1121853 | 6 | 6 | 441721.3 |
| 11734 | Arc | 100000 | Forillon National Park | 875960 | 1721708 | 6 | 6 | 845748.4 |
| 11832 | Arc | 100 | Iles des Rapides de Lachine | 42693 | 80923.36 | 5 | 5 | 38230.36 |
| 11832.001 | Arc | 10000 | Montreal Islands | 70 | 85.18249 | 2 | 2 | 15.18249 |
| 11881.021 | Arc | 1000 | Flowerpot Island | 83.6 | 170.365 | 2 | 2 | 86.76497 |
| 11889 | Arc | 1000000 | Manitoulin Island and adjacent islands | 200 | 255.5475 | 2 | 2 | 55.54746 |
| 11931 | Arc | 1000 | Dorchester Swamp | 479600 | 531794.3 | 6 | 6 | 52194.25 |
| 11949 | Arc | 1000000 | Elgin County | 548 | 2299.927 | 3 | 3 | 1751.927 |
| 11971 | Arc | 1000 | Caughnawaga Indian reservation | 186479 | 213808 | 5 | 5 | 27329.04 |
| 20103 | Atl | 1000 | Experimental watersheds of Hubbard Brook Experimental Forest | 10783 | 31091.61 | 4 | 4 | 20308.61 |
| 20179 | Pac | 10000 | San Bruno Mountains | 36116 | 56646.35 | 5 | 5 | 20530.35 |
| 20508 | Arc | 10000 | Skunk's Misery ANSI | 316278 | 721836.4 | 6 | 6 | 405558.4 |
| 20715 | Atl | 10000000 | West Virginia | 325 | 511.0949 | 3 | 3 | 186.0949 |
| 20766 | Pac | 10000 | Glacial Mountain and vicinity | 6238500 | 20826777 | 7 | 7 | 14588277 |
| 21076 | Atl | 1000 | United Plant Savers Ethnobotanical Sanctuary | 119600 | 147706.4 | 5 | 5 | 28106.43 |
| 21094.002 | Atl | 100 | Site 2 | 48.4 | 85.18249 | 2 | 2 | 36.78249 |
| 21220.001 | Atl | 10000000 | East Slope Central Colorado | 1165495 | 2983602 | 6 | 6 | 1818107 |
| 21255.001 | Pac | 1000 | Bajada Site | 1263400 | 2404105 | 6 | 6 | 1140705 |
| 21268.001 | Arc | 100000 | James Bay Coast | 520 | 1277.737 | 3 | 3 | 757.7373 |
| 21268.008 | Arc | 100000 | Sutton Ridges | 3878900 | 11344603 | 7 | 7 | 7465703 |
| 21275 | Arc | 10000 | Plateau Mountain | 1212173 | 3058562 | 6 | 6 | 1846389 |
| 21701 | Pac | 100 | Fort Clatsop National Memorial | 145300 | 239703.5 | 5 | 5 | 94403.51 |
| 21770 | Atl | 10000 | Camp Beauregard | 1055 | 2981.387 | 3 | 3 | 1926.387 |
| 21836 | Pac | 100 | Irvine Ecological Preserve | 5447 | 25043.65 | 4 | 4 | 19596.65 |
| 21840.005 | Pac | 10000000 | Noatak National Preserve | 3428700 | 13472462 | 7 | 7 | 10043762 |
| 21892 | Atl | 100 | Hancock Biological Station | 34000 | 71553.29 | 5 | 5 | 37553.29 |
| 22080 | Atl | 100000 | Kinzua Quality Deer Cooperative | 129500 | 256228.9 | 5 | 5 | 126728.9 |
| 22263 | Arc | 100 | Cottonwood Lake Study Area | 14575 | 22999.27 | 4 | 4 | 8424.271 |
| 22427 | Pac | 1000 | Area of Lime Creek | 100 | 255.5475 | 2 | 2 | 155.5475 |
| 22488 | Arc | 1000 | Sassafrass Woods Area of Natural and Scientific Interest | 518 | 2299.927 | 3 | 3 | 1781.927 |
| 52 | Pac | 10000000 | Nevada Test Site and Central Southern Nevada | 2590000 | 3477490 | 6 | 7 | 887489.8 |
| 59 | Pac | 1000000 | Uinta Basin | 951138.7 | 7666424 | 6 | 7 | 6715285 |
| 190.02 | Pac | 10000000 | Arctic Slope Foothills | 9242418 | 68951303 | 7 | 8 | 59708885 |
| 237 | Arc | 10000000 | Wager Bay Region | 1950000 | 8066611 | 6 | 7 | 6116611 |
| 265 | Pac | 100 | Onion Peak Preserve | 41.5 | 340.7299 | 2 | 3 | 299.2299 |
| 298 | Pac | 1000000 | Alpine rangelands of Uinta Mountains | 101214.6 | 478470 | 5 | 6 | 377255.4 |
| 334.01 | Pac | 10000 | Great Kobuk and Hunt River Sand Dunes | 6100 | 129903.3 | 4 | 5 | 123803.3 |
| 376.002 | Arc | 1000000 | Bowman County | 301476 | 436134.3 | 5 | 6 | 134658.3 |
| 546 | Arc | 10000 | PattersonHasbala Lakes Region | 3811.36 | 214659.9 | 4 | 5 | 210848.5 |
| 559 | Atl | 1000000 | Chautauqua Hills | 173298 | 646535.1 | 5 | 6 | 473237.1 |
| 645 | Atl | 10000000 | Kansas Flint Hills | 1821862 | 5519825 | 6 | 7 | 3697963 |
| 691 | Arc | 10000 | Goose Mountain Ecological Reserve | 5780 | 60735.11 | 4 | 5 | 54955.11 |
| 712.003 | Pac | 1000 | Batatakin Unit of Navajo National Monument | 183 | 340.7299 | 2 | 3 | 157.7299 |
| 756 | Atl | 1000 | Capulin Volcano National Monument | 275.46 | 1533.285 | 2 | 3 | 1257.825 |
| 786 | Pac | 100000 | Capitol Reef National Park | 97896 | 434430.7 | 5 | 6 | 336534.7 |
| 875 | Pac | 1000000 | part of Kodiak Island | 257500 | 364666.2 | 5 | 6 | 107166.2 |
| 888 | Pac | 1000 | Owyee River Canyon | 777 | 5451.679 | 3 | 4 | 4674.679 |
| 922 | Pac | 100 | Mono Mesa | 64.75 | 340.7299 | 2 | 3 | 275.9799 |
| 1027.002 | Arc | 100 | Mtn. Pk. Townsite | 15 | 85.18249 | 1 | 2 | 70.18249 |
| 1027.006 | Arc | 100 | Mercoal | 12 | 170.365 | 1 | 2 | 158.365 |
| 1133 | Arc | 10000000 | Central Eastern Saskatchewan | 4144000 | 43851943 | 7 | 8 | 39707943 |
| 1294 | Arc | 10000 | Bear's Cove Point Southampton Island | 1818.5 | 7581.241 | 3 | 4 | 5762.741 |
| 1588 | Atl | 10000000 | Rocky Mountains | 1350264 | 4911963 | 6 | 7 | 3561699 |
| 1931 | Arc | 1000000 | Saskatchewan River Delta | 285000 | 1042634 | 5 | 6 | 757633.6 |
| 2011 | Pac | 100000 | Point Reyes National Seashore | 25191 | 100600 | 4 | 5 | 75409 |
| 2493 | Pac | 1000 | Silver Strand Beach State Park | 227 | 700 | 2 | 3 | 473 |
| 2505 | Pac | 1000 | Specimen Creek Research Natural Area | 888 | 3577.664 | 3 | 4 | 2689.664 |
| 2606 | Pac | 10000 | Redwood Regional Park | 1014 | 3407.299 | 3 | 4 | 2393.299 |
| 2666 | Pac | 100000 | Tuolumne Meadows | 17453.17 | 43698.61 | 4 | 5 | 26245.44 |
| 2788 | Pac | 100000 | Bryce Canyon National Park | 14502 | 67464.53 | 4 | 5 | 52962.53 |
| 3331 | Pac | 100 | San Joaquin Freshwater Marsh Reserve | 81.75 | 681.4599 | 2 | 3 | 599.7099 |
| 3424 | Pac | 1000000 | Montane areas of San Diego County | 171000 | 685633.8 | 5 | 6 | 514633.8 |
| 3517.01 | Pac | 100000 | Mount Revelstoke National Park | 26000 | 85863.94 | 4 | 5 | 59863.94 |
| 4026 | Pac | 10000 | Pellisier Flats | 2072 | 20614.16 | 3 | 4 | 18542.16 |
| 5213 | Atl | 1000000 | Cook County | 7812347 | 32462875 | 7 | 8 | 24650528 |
| 10192 | Atl | 100000 | North Carolina Sandhills Wildlife Management Area | 883 | 3407.299 | 3 | 4 | 2524.299 |
| 10283 | Arc | 1000000 | Norfolk County | 25090.5 | 141828.8 | 4 | 5 | 116738.3 |
| 10355.01 | Atl | 10000 | Wallops Island | 176378 | 334682 | 5 | 6 | 158304 |
| 10358 | Atl | 1000 | Indian Bowl Wet Prairie | 1763 | 5800 | 3 | 4 | 4037 |
| 10515 | Atl | 1000000 | Great Smoky Mountains National Park | 105.22 | 340.7299 | 2 | 3 | 235.5099 |
| 10591 | Atl | 10000000 | Collier Dade and Monroe Counties | 210707 | 651986.7 | 5 | 6 | 441279.7 |
| 10821 | Atl | 10000000 | Connecticut | 263661 | 503684 | 5 | 6 | 240023 |
| 11089 | Atl | 100 | Irwin Prairie and adjacent woodland areas | 2260800 | 3742322 | 6 | 7 | 1481522 |
| 11205 | Atl | 10000 | Starved Rock State Park | 77 | 340.7299 | 2 | 3 | 263.7299 |
| 11352 | Atl | 1000000 | Erie County and the peninsula and Islands of Ottawa County | 134 | 1277.737 | 2 | 3 | 1143.737 |
| 11379 | Atl | 1000000 | Lancaster County | 133903 | 327441.5 | 5 | 6 | 193538.5 |
| 11453 | Atl | 100000 | Fakahatchee Strand State Park | 254900 | 631713.3 | 5 | 6 | 376813.3 |
| 11585.001 | Arc | 1000000 | West side of Algonquin Provincial Park | 148 | 1022.19 | 2 | 3 | 874.1898 |
| 11629 | Arc | 10000 | Mont Ste. Marie | 289940 | 577878 | 5 | 6 | 287938 |
| 11690 | Arc | 100 | Isle aux Basques | 800 | 4088.759 | 3 | 4 | 3288.759 |
| 11721 | Arc | 1000000 | Elgin Middlesex and Oxford Counties | 905 | 8433.066 | 3 | 4 | 7528.066 |
| 11725.009 | Arc | 1000000 | Peterborough-Durham-Victoria-Northumberland | 954.16 | 4088.759 | 3 | 4 | 3134.599 |
| 11830 | Arc | 100000 | Diana Bay region | 24035 | 50428.03 | 4 | 5 | 26393.03 |
| 11881.014 | Arc | 100 | Russell Island | 7830 | 225052.1 | 4 | 5 | 217222.1 |
| 20098 | Atl | 100000 | Gateway National Recreation Area | 140 | 1022.19 | 2 | 3 | 882.1898 |
| 20103.007 | Atl | 100 | Watershed 8 | 21.9 | 255.5475 | 1 | 2 | 233.6475 |
| 20121 | Pac | 100000 | Lassen Volcanic National Park | 59.4 | 681.4599 | 2 | 3 | 622.0599 |
| 20195 | Atl | 1000 | Boulder Slope Woods | 1212 | 4173.942 | 3 | 4 | 2961.942 |
| 20324 | Atl | 10000000 | South Central Colorado | 114 | 681.4599 | 2 | 3 | 567.4599 |
| 20506.015 | Arc | 100 | East Sister Island | 1537711 | 4364751 | 6 | 7 | 2827040 |
| 20506.02 | Arc | 100 | Middle Island | 16.6 | 300 | 1 | 2 | 283.4 |
| 20564 | Atl | 10000 | Pecos National Historic Park | 1250 | 4088.759 | 3 | 4 | 2838.759 |
| 20576 | Atl | 100000 | Padre Island National Seashore | 2700 | 11244.09 | 3 | 4 | 8544.088 |
| 20658.007 | Atl | 1000 | Rock Island | 23919 | 37900 | 4 | 5 | 13981 |
| 20823 | Atl | 100000 | Mathews County | 10000 | 35435.91 | 4 | 5 | 25435.91 |
| 20963 | Pac | 1000 | Red Rock State Park | 27194.88 | 46100 | 4 | 5 | 18905.13 |
| 20971 | Pac | 100 | West Branch Study Area | 116 | 511.0949 | 2 | 3 | 395.0949 |
| 21087 | Atl | 100 | Madeline Bertrand Park | 155 | 681.4599 | 2 | 3 | 526.4599 |
| 21123 | Atl | 10000000 | Upper Green River Basin | 14.8 | 200 | 1 | 2 | 185.2 |
| 21299 | Atl | 1000 | Panther Rock | 1500 | 6814.599 | 3 | 4 | 5314.599 |
| 21310 | Atl | 10000 | Piscataway and Fort Washington National Parks | 202 | 1022.19 | 2 | 3 | 820.1898 |
| 21312 | Atl | 100 | Dean Hills Nature Preserve | 1956 | 8347.884 | 3 | 4 | 6391.884 |
| 21370 | Atl | 100000 | Camp Gruber | 30 | 255.5475 | 1 | 2 | 225.5475 |
| 21450 | Pac | 10000000 | Denali National Park and Preserve | 19500 | 47020.73 | 4 | 5 | 27520.73 |
| 21528 | Atl | 100 | Winous Point and Squaw Island | 2458500 | 8812639 | 6 | 7 | 6354139 |
| 21562 | Arc | 10000 | Mount Yamaska | 32.3 | 2129.562 | 2 | 3 | 2097.262 |
| 21594 | Pac | 100000 | San Pedro Riparian National Conservation Area | 1256 | 5366.497 | 3 | 4 | 4110.497 |
| 21599 | Atl | 10000 | Plum Island | 19291 | 134929.1 | 4 | 5 | 115638.1 |
| 21614 | Atl | 1000000 | Jasper County | 1475 | 5300 | 3 | 4 | 3825 |
| 21731 | Atl | 10000 | Strouds Run State Park | 50.6 | 511.0949 | 2 | 3 | 460.4949 |
| 21840.003 | Pac | 10000000 | Gates of the Arctic National Park and Preserve | 25 | 170.365 | 1 | 2 | 145.365 |
| 21869 | Pac | 100000 | Kingston Range | 2658700 | 14359297 | 6 | 7 | 11700597 |
| 21937 | Pac | 1000 | Mount St. Helens Crater | 37.5 | 340.7299 | 2 | 3 | 303.2299 |
| 21939 | Pac | 10000 | West Fork of Oak Creek Canyon | 314 | 1022.19 | 2 | 3 | 708.1898 |
| 22008 | Pac | 1000000 | Whipple Mountains | 2040 | 12777.37 | 3 | 4 | 10737.37 |
| 22095 | Pac | 10000000 | Lake Clark National Park and Preserve | 30628 | 91997.08 | 4 | 5 | 61369.08 |
| 22134 | Pac | 1000 | Caswell Memorial State Park | 1630800 | 5129860 | 6 | 7 | 3499060 |
| 22135 | Atl | 100 | Wallace Woods | 104 | 1022.19 | 2 | 3 | 918.1898 |
| 22249 | Atl | 100000 | Chama Land and Cattle Company and Frank Sims Ranch | 12.9 | 170.365 | 1 | 2 | 157.465 |
| 22279 | Arc | 1000 | Wainfleet Bog ANSI | 92 | 425.9124 | 2 | 3 | 333.9124 |
| 22363 | Arc | 1000 | Royal Botanical Gardens Nature Sanctuaries | 251 | 1022.19 | 2 | 3 | 771.1898 |
| 22363.002 | Arc | 100 | Hendrie Valley | 805 | 5962.774 | 3 | 4 | 5157.774 |
| 22493 | Arc | 100000 | Proposed Parc National du Lac-Temiscouata | 136 | 1022.19 | 2 | 3 | 886.1898 |
| 22518.007 | Pac | 100 | Rocky | 21300 | 87226.86 | 4 | 5 | 65926.86 |
| 22518.011 | Pac | 100 | Tenalquot | 12 | 255.5475 | 1 | 2 | 243.5475 |
| 22518.012 | Pac | 10000 | 13th Division | 38 | 511.0949 | 2 | 3 | 473.0949 |
| 22521 | Atl | 10000 | Chattahoochie River National Recreation Area | 1114 | 5110.949 | 3 | 4 | 3996.949 |
| 22651 | Arc | 10000 | Stony Swamp Conservation Area | 1237 | 3577.664 | 3 | 4 | 2340.664 |
| 22621 | Arc | 10000 | Charleston Lake Provincial Park | 1900 | 7496.059 | 3 | 4 | 5596.059 |
| 132 | Atl | 100 | Brayton Horsley Prairie | 14 | 511.0949 | 1 | 3 | 497.0949 |
| 203 | Arc | 100000 | Nahanni National Park and vicinity | 70722.6 | 5651688 | 5 | 7 | 5580965 |
| 1027.004 | Arc | 100 | Nordegg | 12 | 1788.832 | 1 | 3 | 1776.832 |
| 2457 | Pac | 100 | Pixley Preserve | 16.2 | 340.7299 | 1 | 3 | 324.5299 |
| 10779 | Atl | 1000000 | Bedford County | 439 | 51109.49 | 3 | 5 | 50670.49 |
| 11701 | Arc | 1000 | Niagara Escarpment and Grindstone Creek Public Open Space | 55 | 3236.934 | 2 | 4 | 3181.934 |
| 20103.005 | Atl | 100 | Watershed 5 | 261 | 4599.854 | 2 | 4 | 4338.854 |
| 20506.024 | Atl | 1000000 | Erie Ottawa and Essex Counties | 19.88 | 400 | 1 | 3 | 380.12 |
| 21025 | Atl | 1000000 | Oktibbeha County | 29 | 340.7299 | 1 | 3 | 311.7299 |
| 21268.006 | Arc | 10000000 | Albany Basin | 66763 | 4494569 | 5 | 7 | 4427806 |
| 21268.009 | Arc | 10000000 | Cape Henrietta Maria | 20000 | 3116401 | 4 | 6 | 3096401 |
| 22633 | Pac | 10000 | Daley Ranch | 2618 | 110396.5 | 3 | 5 | 107778.5 |

*Notes*: Data are arranged first by order of magnitude difference between flora and bounding box and then by flora accession number. Colors indicate orders of magnitude difference: green=0, yellow=1, orange=2. Flora accession number can be referenced for additional information and citation in Appendix S1. Drainage basins are abbreviated as Atlantic (Atl), Arctic (Arc), and Pacific (Pac).
