# Supplementary material for: How does spatial extent and environmental limits affect the accuracy of species richness estimates from ecological niche models? A case study with North American Pinaceae and Cactaceae
Source: Ecol Evol. 2023 Apr 21;13(4):e10007. doi: 10.1002/ece3.10007 (PMC10121319; doi:10.1002/ece3.10007)
Supplement: Supplementary file 6 — Table S2: [file ECE3-13-e10007-s007.docx]

**Table S2: S**tratification of sampling of floras from the Floras of North America project Database.

| **Drainage Region** | **Size classes of geographic areas in hectares** | | | | | | |  |
| --- | --- | --- | --- | --- | --- | --- | --- | --- |
|  | **10^1^*<x≤*10^2^** | **10^2^*<x≤*10^3^** | **10^3^*<x≤*10^4^** | **10^4^*<x≤*10^5^** | **10^5^*<x≤*10^6^** | **10^6^*<x≤*10^7^** |  |  |
| **Arctic** | 10 | 10 | 10 | 10 | 10 | 10 | **60** |  |
| **Atlantic** | 10 | 10 | 10 | 10 | 10 | 10 | **60** |  |
| **Pacific** | 10 | 10 | 10 | 10 | 10 | 10 | **60** |  |
| **Totals** | **30** | **30** | **30** | **30** | **30** | **30** | **180** |  |

*Notes*: Sizes of geographic areas given in hectares. States of the United States, Canadian provinces, and the French territory of Saint Pierre and Miquelon have been divided into three groups depending on the primary basin to which their freshwater drains as follows: **Arctic** (Alberta, Manitoba, North Dakota, Northwest Territories, Nunavut, Ontario, Quebec, Saskatchewan), **Atlantic** (Alabama, Arkansas, Colorado, Connecticut, Delaware, Florida, Georgia, Illinois, Indiana, Iowa, Kansas, Kentucky, Louisiana, Maine, Maryland, Massachusetts, Michigan, Minnesota, Mississippi, Missouri, Montana, Nebraska, New Brunswick, New Hampshire, New Jersey, New Mexico, New York, Newfoundland and Labrador, North Carolina, Nova Scotia, Ohio, Oklahoma, Pennsylvania, Prince Edward Island, Rhode Island, Saint Pierre and Miquelon, South Carolina, South Dakota, Tennessee, Texas, Vermont, Virginia, Washington DC, West Virginia, Wisconsin, Wyoming), and **Pacific** (Alaska, Arizona, British Columbia, California, Hawaii, Idaho, Nevada, Oregon, Utah, Washington, Yukon).
