# Supplementary material for: How does spatial extent and environmental limits affect the accuracy of species richness estimates from ecological niche models? A case study with North American Pinaceae and Cactaceae
Source: Ecol Evol. 2023 Apr 21;13(4):e10007. doi: 10.1002/ece3.10007 (PMC10121319; doi:10.1002/ece3.10007)
Supplement: Supplementary file 7 — Table S3: [file ECE3-13-e10007-s005.docx]

**Table S2:** Number of occurrences used for modeling each species of Cactaceae and Pinaceae

| **Species** | **Family** | **Category** | **Number Occurrences for Modeling** |
| --- | --- | --- | --- |
| *Consolea corallicola* | Cactaceae | insufficient records for modeling | 0 |
| *Cylindropuntia kelvinensis* | Cactaceae | insufficient records for modeling | 0 |
| *Cylindropuntia tetracantha* | Cactaceae | insufficient records for modeling | 0 |
| *Harrisia aboriginum* | Cactaceae | insufficient records for modeling | 0 |
| *Opuntia cubensis* | Cactaceae | insufficient records for modeling | 0 |
| *Opuntia triacantha* | Cactaceae | insufficient records for modeling | 0 |
| *Pediocactus winkleri* | Cactaceae | insufficient records for modeling | 0 |
| *Pereskia aculeata* | Cactaceae | insufficient records for modeling | 0 |
| *Pilosocereus robinii* | Cactaceae | insufficient records for modeling | 0 |
| *Sclerocactus whipplei* | Cactaceae | insufficient records for modeling | 0 |
| *Harrisia simpsonii* | Cactaceae | insufficient records for modeling | 6 |
| *Opuntia monacantha* | Cactaceae | insufficient records for modeling | 3 |
| *Acanthocereus tetragonus* | Cactaceae | records sufficient for model building | 41 |
| *Bergerocactus emoryi* | Cactaceae | records sufficient for model building | 23 |
| *Brasiliopuntia brasiliensis* | Cactaceae | records sufficient for model building | 77 |
| *Carnegiea gigantea* | Cactaceae | records sufficient for model building | 83 |
| *Coryphantha missouriensis* | Cactaceae | records sufficient for model building | 70 |
| *Cylindropuntia arbuscula* | Cactaceae | records sufficient for model building | 75 |
| *Cylindropuntia bigelovii* | Cactaceae | records sufficient for model building | 31 |
| *Cylindropuntia californica* | Cactaceae | records sufficient for model building | 79 |
| *Cylindropuntia kleiniae* | Cactaceae | records sufficient for model building | 30 |
| *Cylindropuntia prolifera* | Cactaceae | records sufficient for model building | 47 |
| *Cylindropuntia versicolor* | Cactaceae | records sufficient for model building | 41 |
| *Echinocactus polycephalus* | Cactaceae | records sufficient for model building | 63 |
| *Echinocereus coccineus* | Cactaceae | records sufficient for model building | 83 |
| *Echinocereus fasciculatus* | Cactaceae | records sufficient for model building | 75 |
| *Echinocereus pectinatus* | Cactaceae | records sufficient for model building | 39 |
| *Echinocereus rigidissimus* | Cactaceae | records sufficient for model building | 29 |
| *Echinocereus yavapaiensis* | Cactaceae | records sufficient for model building | 7 |
| *Echinomastus intertextus* | Cactaceae | records sufficient for model building | 35 |
| *Ferocactus viridescens* | Cactaceae | records sufficient for model building | 18 |
| *Ferocactus wislizenii* | Cactaceae | records sufficient for model building | 89 |
| *Grusonia emoryi* | Cactaceae | records sufficient for model building | 31 |
| *Grusonia parishii* | Cactaceae | records sufficient for model building | 39 |
| *Grusonia pulchella* | Cactaceae | records sufficient for model building | 18 |
| *Mammillaria dioica* | Cactaceae | records sufficient for model building | 46 |
| *Mammillaria heyderi* | Cactaceae | records sufficient for model building | 61 |
| *Mammillaria macdougalii* | Cactaceae | records sufficient for model building | 8 |
| *Mammillaria tetrancistra* | Cactaceae | records sufficient for model building | 38 |
| *Mammillaria thornberi* | Cactaceae | records sufficient for model building | 16 |
| *Mammillaria wrightii* | Cactaceae | records sufficient for model building | 45 |
| *Opuntia aciculata* | Cactaceae | records sufficient for model building | 8 |
| *Opuntia ficus-indica* | Cactaceae | records sufficient for model building | 38 |
| *Opuntia littoralis* | Cactaceae | records sufficient for model building | 65 |
| *Opuntia macrocentra* | Cactaceae | records sufficient for model building | 93 |
| *Opuntia occidentalis* | Cactaceae | records sufficient for model building | 7 |
| *Opuntia oricola* | Cactaceae | records sufficient for model building | 25 |
| *Opuntia pottsii* | Cactaceae | records sufficient for model building | 29 |
| *Opuntia stricta* | Cactaceae | records sufficient for model building | 20 |
| *Opuntia vaseyi* | Cactaceae | records sufficient for model building | 36 |
| *Pediocactus simpsonii* | Cactaceae | records sufficient for model building | 91 |
| *Peniocereus greggii* | Cactaceae | records sufficient for model building | 60 |
| *Sclerocactus parviflorus* | Cactaceae | records sufficient for model building | 61 |
| *Sclerocactus polyancistrus* | Cactaceae | records sufficient for model building | 17 |
| *Coryphantha vivipara* | Cactaceae | records sufficient for model building and testing | 336 |
| *Cylindropuntia acanthocarpa* | Cactaceae | records sufficient for model building and testing | 232 |
| *Cylindropuntia echinocarpa* | Cactaceae | records sufficient for model building and testing | 161 |
| *Cylindropuntia fulgida* | Cactaceae | records sufficient for model building and testing | 102 |
| *Cylindropuntia imbricata* | Cactaceae | records sufficient for model building and testing | 133 |
| *Cylindropuntia leptocaulis* | Cactaceae | records sufficient for model building and testing | 266 |
| *Cylindropuntia ramosissima* | Cactaceae | records sufficient for model building and testing | 101 |
| *Cylindropuntia spinosior* | Cactaceae | records sufficient for model building and testing | 160 |
| *Cylindropuntia whipplei* | Cactaceae | records sufficient for model building and testing | 145 |
| *Echinocereus englemannii* | Cactaceae | records sufficient for model building and testing | 278 |
| *Echinocereus fendleri* | Cactaceae | records sufficient for model building and testing | 108 |
| *Echinocereus triglochidiatus* | Cactaceae | records sufficient for model building and testing | 265 |
| *Echinocereus viridiflorus* | Cactaceae | records sufficient for model building and testing | 123 |
| *Ferocactus cylindraceus* | Cactaceae | records sufficient for model building and testing | 138 |
| *Mammillaria grahamii* | Cactaceae | records sufficient for model building and testing | 125 |
| *Opuntia basilaris* | Cactaceae | records sufficient for model building and testing | 216 |
| *Opuntia chlorotica* | Cactaceae | records sufficient for model building and testing | 138 |
| *Opuntia engelmannii* | Cactaceae | records sufficient for model building and testing | 261 |
| *Opuntia fragilis* | Cactaceae | records sufficient for model building and testing | 161 |
| *Opuntia humifusa* | Cactaceae | records sufficient for model building and testing | 173 |
| *Opuntia macrorhiza* | Cactaceae | records sufficient for model building and testing | 375 |
| *Opuntia phaeacantha* | Cactaceae | records sufficient for model building and testing | 433 |
| *Opuntia polyacantha* | Cactaceae | records sufficient for model building and testing | 481 |
| *Rhipsalis baccifera* | Cactaceae | records sufficient for model building and testing | 126 |
| *Pinus cedrus* | Pinaceae | insufficient records for modeling | 0 |
| *Abies bifolia* | Pinaceae | records sufficient for model building | 66 |
| *Abies fraseri* | Pinaceae | records sufficient for model building | 64 |
| *Pinus clausa* | Pinaceae | records sufficient for model building | 91 |
| *Pinus elliottii* | Pinaceae | records sufficient for model building | 92 |
| *Pinus pumila* | Pinaceae | records sufficient for model building | 11 |
| *Pinus pungens* | Pinaceae | records sufficient for model building | 62 |
| *Pinus serotina* | Pinaceae | records sufficient for model building | 52 |
| *Pinus sylvestris* | Pinaceae | records sufficient for model building | 39 |
| *Pinus thunbergii* | Pinaceae | records sufficient for model building | 65 |
| *Abies amabilis* | Pinaceae | records sufficient for model building and testing | 216 |
| *Abies balsamea* | Pinaceae | records sufficient for model building and testing | 829 |
| *Abies concolor* | Pinaceae | records sufficient for model building and testing | 894 |
| *Abies grandis* | Pinaceae | records sufficient for model building and testing | 360 |
| *Abies lasiocarpa* | Pinaceae | records sufficient for model building and testing | 343 |
| *Abies magnifica* | Pinaceae | records sufficient for model building and testing | 301 |
| *Abies procera* | Pinaceae | records sufficient for model building and testing | 164 |
| *Larix decidua* | Pinaceae | records sufficient for model building and testing | 3591 |
| *Larix laricina* | Pinaceae | records sufficient for model building and testing | 856 |
| *Picea abies* | Pinaceae | records sufficient for model building and testing | 55601 |
| *Picea engelmannii* | Pinaceae | records sufficient for model building and testing | 518 |
| *Picea glauca* | Pinaceae | records sufficient for model building and testing | 1034 |
| *Picea mariana* | Pinaceae | records sufficient for model building and testing | 1131 |
| *Picea pungens* | Pinaceae | records sufficient for model building and testing | 172 |
| *Picea sitchensis* | Pinaceae | records sufficient for model building and testing | 275 |
| *Pinus albicaulis* | Pinaceae | records sufficient for model building and testing | 435 |
| *Pinus aristata* | Pinaceae | records sufficient for model building and testing | 177 |
| *Pinus attenuata* | Pinaceae | records sufficient for model building and testing | 405 |
| *Pinus banksiana* | Pinaceae | records sufficient for model building and testing | 389 |
| *Pinus cembroides* | Pinaceae | records sufficient for model building and testing | 527 |
| *Pinus contorta* | Pinaceae | records sufficient for model building and testing | 1092 |
| *Pinus coulteri* | Pinaceae | records sufficient for model building and testing | 318 |
| *Pinus echinata* | Pinaceae | records sufficient for model building and testing | 326 |
| *Pinus edulis* | Pinaceae | records sufficient for model building and testing | 737 |
| *Pinus engelmannii* | Pinaceae | records sufficient for model building and testing | 209 |
| *Pinus flexilis* | Pinaceae | records sufficient for model building and testing | 742 |
| *Pinus halepensis* | Pinaceae | records sufficient for model building and testing | 19921 |
| *Pinus jeffreyi* | Pinaceae | records sufficient for model building and testing | 501 |
| *Pinus lambertiana* | Pinaceae | records sufficient for model building and testing | 461 |
| *Pinus leiophylla* | Pinaceae | records sufficient for model building and testing | 513 |
| *Pinus longaeva* | Pinaceae | records sufficient for model building and testing | 210 |
| *Pinus monophylla* | Pinaceae | records sufficient for model building and testing | 761 |
| *Pinus monticola* | Pinaceae | records sufficient for model building and testing | 528 |
| *Pinus mugo* | Pinaceae | records sufficient for model building and testing | 181 |
| *Pinus muricata* | Pinaceae | records sufficient for model building and testing | 424 |
| *Pinus palustris* | Pinaceae | records sufficient for model building and testing | 163 |
| *Pinus ponderosa* | Pinaceae | records sufficient for model building and testing | 1469 |
| *Pinus quadrifolia* | Pinaceae | records sufficient for model building and testing | 242 |
| *Pinus radiata* | Pinaceae | records sufficient for model building and testing | 217 |
| *Pinus resinosa* | Pinaceae | records sufficient for model building and testing | 250 |
| *Pinus rigida* | Pinaceae | records sufficient for model building and testing | 212 |
| *Pinus strobiformis* | Pinaceae | records sufficient for model building and testing | 194 |
| *Pinus strobus* | Pinaceae | records sufficient for model building and testing | 566 |
| *Pinus taeda* | Pinaceae | records sufficient for model building and testing | 213 |
| *Pinus torreyana* | Pinaceae | records sufficient for model building and testing | 121 |
| *Pinus virginiana* | Pinaceae | records sufficient for model building and testing | 180 |
| *Pseudotsuga macrocarpa* | Pinaceae | records sufficient for model building and testing | 294 |
| *Pseudotsuga menziesii* | Pinaceae | records sufficient for model building and testing | 1214 |
| *Tsuga canadensis* | Pinaceae | records sufficient for model building and testing | 541 |
| *Tsuga heterophylla* | Pinaceae | records sufficient for model building and testing | 396 |
| *Tsuga mertensiana* | Pinaceae | records sufficient for model building and testing | 511 |
