# Supplementary material for: How does spatial extent and environmental limits affect the accuracy of species richness estimates from ecological niche models? A case study with North American Pinaceae and Cactaceae
Source: Ecol Evol. 2023 Apr 21;13(4):e10007. doi: 10.1002/ece3.10007 (PMC10121319; doi:10.1002/ece3.10007)
Supplement: Supplementary file 8 — Table S4: [file ECE3-13-e10007-s002.docx]

**Table S4:** Illustration of a basic confusion matrix.

|  |  | **Floras**  **(Reported)** | |
| --- | --- | --- | --- |
|  |  | **Present** | **Absent** |
| **Stacked Ecological Niche Models**  **(Predicted)** | **Present** | a | b |
|  | **Absent** | c | d |

*Notes*: The variables a-d represent the four possible relationships between a model prediction for a taxon and its reported (or reported, true) presence or absence from a geographic area or, in this case, from a flora. Sensitivity was calculated as a/(a+c) and specificity was b/(b+d).
